# Aniline–phenol recognition: from solution through supramolecular synthons to cocrystals

**DOI:** 10.1107/S2052252514012081

**Published:** 2014-06-12

**Authors:** Arijit Mukherjee, Karuna Dixit, Siddhartha P. Sarma, Gautam R. Desiraju

**Affiliations:** a Solid State and Structural Chemistry Unit, Indian Institute of Science, Bangalore 560 012, India; b Molecular Biophysics Unit, Indian Institute of Science, Bangalore 560 012, India

**Keywords:** supramolecular synthon, crystal engineering, cocrystal, crystal structure prediction

## Abstract

Aniline–phenol recognition is studied in the crystal engineering context in several 1:1 cocrystals that contain a closed cyclic hydrogen-bonded [⋯O—H⋯N—H⋯]_2_ tetramer supramolecular synthon (**II**). Twelve cocrystals of 3,4,5- and 2,3,4-trichlorophenol with one of eight halogenated anilines have been characterized. Ten of these cocrystals contain an extended octamer synthon that is assembled with hydrogen bonding and π⋯π stacking that defines a *Long-Range Synthon Aufbau Module* (LSAM). The design strategy is, therefore, based on the construction and transferability of the LSAM, which is a dimer of tetramers. Using the LSAM concept, two short cell axes in the crystal structures can be predicted. Whilst one of them is dictated by synthon **II**, the other one is dominated by π⋯π interactions. The third cell axis can also be predicted, in some cases, by systematic tuning of the halogen bonds. The design strategy is also verified in cocrystals of non-halogenated precursors. The observation of this large synthon in so many structures points to its stability and possible existence in solution. To this end, one-dimensional ^1^H and ^15^N NMR studies, performed on the 3,4,5-trichlorophenol–3,5-dichloroaniline cocrystal in CDCl_3_, show characteristic downfield shifts that point to a π⋯π stacked structure and to the robustness of the hydrogen-bonded aggregates. Nuclear Overhauser effects point to hydrogen bonding between aniline and phenol molecules in the aggregates. Diffusion-ordered spectroscopy and *T*_1_ inversion recovery experiments show that stacking is present in concentrated solution and lost at a certain dilution. A sequence of events is therefore established: molecules of the aniline and the phenol associate *via* hydrogen bonding to form tetramers, and tetramers subsequently stack to form octamers.

## Abstract 2

The Long-Range Synthon Aufbau Module (LSAM) concept is utilized in the crystal engineering of 1:1 cocrystals of trichlorophenols and halogen-substituted anilines. NMR studies show the presence of LSAMs in solution and also the sequence of association of hydrogen bonding and π⋯π stacking interations that constitute the LSAMs.

## 1. Introduction   

The study of aniline–phenol recognition, in the context of crystal engineering and supramolecular synthons, has been an unusually complex exercise, considering the small size and relative simplicity of the —NH_2_ and —OH functional groups. At the first level, only the hydrogen-bonding capabilities of the amino and hydroxyl groups appear to be important; Ermer & Eling (1994 ▶) and Hanessian *et al.* (1994 ▶, 1995 ▶, 1999 ▶) independently predicted that in systems with an equal stoichiometry of —OH and —NH_2_ groups, there are equal numbers of O—H⋯N and N—H⋯O hydrogen bonds, leading to tetrahedral configurations at both N and O atoms. Allen *et al.* (1997 ▶) showed, however, that this seemingly simple model, which was applied to 4-aminophenol by Ermer, ignores the herringbone interactions of the phenyl rings, as seen in the N—H⋯π-based structures of 2- and 3-aminophenol. The appearance of N—H⋯π interactions in many of these structures is a manifestation of interference between hydrocarbon residues and the hydrogen-bonding groups (Desiraju, 2001 ▶). This is a real issue in many aminophenols. Vangala *et al.* (2003 ▶) demonstrated that 3-aminophenol is a prototype and that an entire family of methylene-linked aminophenols can be understood as a balance between the infinite ⋯O—H⋯N—H⋯ synthon and various hydrocarbon interactions, including the non-conventional N—H⋯π hydrogen bond. Also described by them is the concept of synthon evolution which may be examined in systems of large enough size and complexity. Dey *et al.* (2005 ▶) hinted at the importance of large synthons (containing both hydrogen bonds and C—H⋯π herringbone interactions) in the context of crystallization mechanisms in a crystal structure prediction of isomeric methyl aminophenols. The conjoining of hydrogen bonds and hydrocarbon interactions leads to an increase in both complexity and size of certain important synthons in this system and these have been referred to as *Long Range Synthon Aufbau Modules* (LSAM) by Ganguly & Desiraju (2010 ▶). These guidelines apply equally well to multi-component systems: Vangala *et al.* (2004 ▶) studied a series of dianiline–diphenol molecular complexes or cocrystals in this regard. Desiraju (2013 ▶) suggested recently that the so-called large synthons or LSAMs in the aminophenols could be a sought-after bridge between small synthons and crystal growth units.

Intermolecular association and aggregation in solution may be probed by NMR spectroscopic methods in a facile manner (Spitaleri *et al.*, 2004 ▶; Chiarella *et al.*, 2007 ▶; Chadwick *et al.*, 2009 ▶; Schneider, 2009 ▶). The most direct evidence for molecular association/aggregation comes from perturbations in chemical shifts (δ) between the free and the associated form of interacting solutes (Saito *et al.*, 2002 ▶; Spitaleri *et al.*, 2004 ▶). Perturbations in chemical shifts arise from differences in the magnetic environment that the nuclear spins experience in the free and associated forms. Molecular association may also be inferred from other well established NMR parameters such as the spin-lattice relaxation time constants (*T*_1_) (a measure of the time required for the nuclear spins under investigation to return to thermal equilibrium after a perturbation) (Claridge, 2008 ▶) and from estimates of translational diffusion coefficients (*D*_s_) (Diffusion Ordered Spectroscopy, DOSY, is an experimental method designed to estimate the translational diffusion coefficient of solutes) (Barjat *et al.*, 1995 ▶; Cohen *et al.*, 2005 ▶). The magnitude of Nuclear Overhauser effect (NOE) enhancement is dependent on the fact that the distance of separation between the nuclear magnetic moments must lie within ∼5 Å (Neuhaus & Williamson, 2000 ▶). Fortunately, the weak forces that stabilize intermolecular interactions such as hydrogen bonding, van der Waals interactions, hydrophobic interactions or salt bridges act over short distances. Thus, the existence of intermolecular associations may be established by assignment of the NOE between the interacting species. The longitudinal relaxation rates are sensitive to the rotational correlation time (τ_c_) and thus proportional to the molecular size. Similarly, the translational diffusion coefficient is also proportional to the molecular size. A significant advantage of solution NMR methods lies in their ability to explore association/aggregation properties of solutes as a function of solute concentration, and whether or not association/aggregation occurs.

With this background, we initiated a study of the crystal chemistry of a series of phenol–aniline cocrystals based mostly on 3,4,5-trichlorophenol, **1**, and halogenated anilines, **3**–**10**. Phenol **1** has a highly modular crystal structure that may be developed (unusually) from the crystal structures of 4-chloro­phenol and 3,5-dichlorophenol (Mukherjee & Desiraju, 2011 ▶). It must be noted here that modularity is an inherent property of supramolecular synthons and, in that sense, every crystal structure has certain modular features. However, as weak interactions play a major role at the end stages of crystallization, this modularity is often restricted to the primary synthon level. The unusual feature of phenol **1** is that it shows the rare phenomenon of modularity at a large synthon level. This type of modularity is indicative of high structural insulation, which is useful for the type of study performed here. The family of structures studied here, therefore, lends itself particularly well to the principles of crystal engineering and also provides an opportunity for the study of large synthons in solution and their evaluation as intermediates during crystallization.[Chem scheme1] 
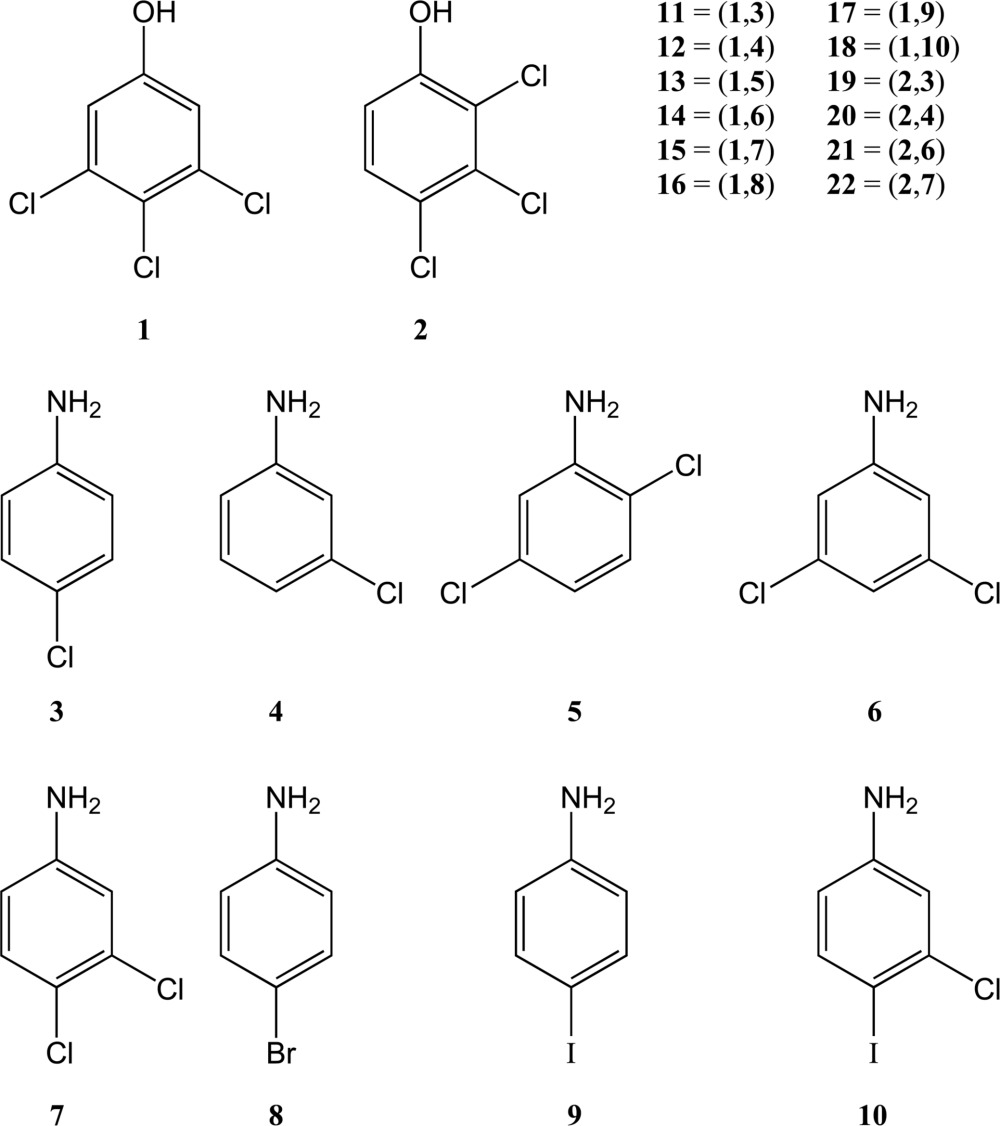


## 2. Experimental   

### 2.1. Synthesis and single-crystal X-ray diffraction   

The cocrystals were prepared generally *via* solvent drop grinding (see supporting information for details), except those with 3-chloroaniline, which is a liquid at ambient conditions. Single crystals were obtained mostly by solvent evaporation except in a few cases where crystals were obtained by sublimation. The details of the crystallization procedure for each cocrystal are given in the supporting information. After obtaining crystals of suitable size and quality, single-crystal X-ray data were collected on a Rigaku Mercury375R/M CCD (XtaLAB mini) diffractometer using graphite-monochromated Mo *K*α radiation. The instrument was attached to a Rigaku low-temperature gas-spray cooler. The data were processed with the *CrystalClear* software (Rigaku, 2009 ▶). Structure solution and refinements were performed using *SHELX*97 incorporated within the *WinGX* suite (Sheldrick, 2008 ▶; Farrugia, 1999 ▶).

### 2.2. Database studies   

A search of the Cambridge Structural Database (CSD, Version 5.34, with updates to May 2013; Allen, 2002 ▶) was performed to find crystal structures which contain both aniline and phenol residues. The search was restricted according to the following criteria: three-dimensional structures determined, *R* < 0.075, not disordered, no errors, not polymeric, no ions, no powder structures, only organics. The resulting structures were analysed manually to identify synthon patterns and their relative frequencies.

### 2.3. NMR spectroscopic techniques   

NMR spectra were acquired on Bruker 400 or 500 MHz NMR spectrometers or on an Agilent 600 MHz NMR spectrometer. All spectra were acquired at 298 K. A ^1^H spectral width of 10 p.p.m. was sampled at all field strengths. A ^13^C spectral width of 100 p.p.m. was sampled at all field strengths. One-dimensional ^15^N spectra were recorded at 600 MHz at natural abundance, using a spectral width of 12 019 Hz. Urea (BrukerBiospin Standard, 0.1 *M* ^15^N-urea in dimethyl sulfoxide) was taken as a reference compound. A total of 64, 8192, 16 384 and 81 920 transients were recorded for the urea reference, and for samples **A**, **E** and **F** (see below). During acquisition, a 5 kHz field was applied to achieve proton decoupling. ^15^N chemical shifts are referenced to external DSS (Cavanagh *et al.*, 2007 ▶). ^1^H and ^13^C chemical shifts are referenced to internal TMS (0.0 p.p.m.) and CDCl_3_ (solvent, 77 p.p.m.), respectively.

#### 2.3.1. Sample preparation   

3,4,5-Trichlorophenol and 3,5-dichloroaniline were taken in a 1:1 molar ratio and ground together in a mortar to obtain cocrystal **14**. The resulting powder was dissolved in CDCl_3_ to obtain a concentration of 1.25 *M*. This concentration is taken as the starting point in the dilution study and is called **A**. This solution was then diluted gradually from **A** to **B** (1 *M*) to **C** (0.8 *M*) to **D** (0.7 *M*) to **E** (0.625 *M*) and finally to **F** (0.125 *M*). Solutions of 1,2,3-trichlorobenzene were prepared in a similar manner from a stock solution (1.25 *M*) and labelled **A_1_** to **F_1_**.

#### 2.3.2. Chemical shift perturbation   

One-dimensional NMR spectra of solutions **A** to **F** (**14**) and **A_1_** to **F_1_** (1,2,3-trichloro­benzene) were recorded on the Bruker 400 MHz NMR spectrometer using a BBI probe fitted with a single (*z*-axis) pulsed field gradient (PFG) accessory. All spectra were processed using *TopSpin 3.2* software (Bruker, 2014 ▶).

#### 2.3.3. Estimation of longitudinal relaxation time constants (*T*_1_)   

Data for *T*_1_ estimation of ^13^C in samples of **A**, **E** and **F** (**14**) and **A_1_**, **E_1_** and **F_1_** (1,2,3-trichlorobenzene) were acquired using the *T*_1_ inversion recovery method (Claridge, 2008 ▶). Data were acquired on the Bruker 500 MHz spectrometer using a TXI probe fitted with a *z*-axis PFG accessory and the Agilent 600 MHz NMR spectrometer using an IDTRPFG probe fitted with a *z*-axis gradient accessory. Interscan delays of 15 and 25 s were maintained at 500 and 600 MHz, respectively. ^13^C *T*_1_ values were measured from spectra recorded with 25 different durations of the recovery delay at 500 MHz: τ = 50, 100, 500 ms, 1.0–8.0 s in steps of 0.5, 9, 10, 12, 15, 18, 21 and 25 s. *T*_1_ values at 600 MHz were measured from 13 different durations of the recovery delay for sample **A**: τ = 100, 500 ms, 1.0–5.0 s in steps of 0.5, 7.5 and 10 s; and from 11 different durations of the recovery delay for samples **E** and **F**: τ = 0.1, 0.5, 1, 2, 4, 6, 8, 10, 12.5, 15 and 20 s. Spectra were processed using the *VnmrJ 3.2A* software (Agilent, 2012 ▶). The peak heights were fitted to the equation
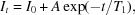
where *I_t_* is the peak height at time *t*, *I*_0_ is the peak height at *t* = 0 and *A* is a constant. *T*_1_ values were calculated using peak-fitting routines in *VnmrJ 3.2A*.

#### 2.3.4. Difference NOE experiments   

Difference NOE spectra (Neuhaus & Williamson, 2000 ▶) on solutions **A**, **E** and **F** (**14**) and **A_1_**, **E_1_** and **F_1_** (1,2,3-trichlorobenzene) were acquired on the Agilent 600 MHz NMR spectrometer using the IDTRPFG probe fitted with a *z*-axis PFG accessory. Low power saturation was applied for 3 s followed by data acquisition. In the case of the no-saturation experiment, the transmitter was placed at −2 p.p.m. A relaxation delay of 25 s was maintained between transients.

#### 2.3.5. Estimation of translational diffusion coefficients   

Translational diffusion coefficients were measured using the one-dimensional DOSY bipolar pulsed pair gradient stimulated echo experiment (Stejskal & Tanner, 1965 ▶; Johnson, 1999 ▶) on the Agilent 600 MHz NMR spectrometer. All spectra were acquired using the IDTRPFG probe fitted with a *z*-axis PFG accessory. Data were acquired at three values of the diffusion delay (75, 85 and 100 ms) for gradient strengths (G cm^−1^) of 2.10, 2.73, 4.62, 10.07, 18.55, 25.02, 30.60, 35.29, 39.43, 43.18, 46.63, 49.84, 52.85, 55.70, 58.41, 61.00, 63.49, 65.88 and 68.20. A relaxation delay of 25 s was maintained between successive transients. Bipolar pulsed field gradients were applied for a total duration of 2 ms and a gradient recovery delay time of 500 µs introduced prior to application of RF pulses. Spectra were processed using *VnmrJ 32A* software. The translational diffusion coefficient (*D*) was obtained by fitting to the equation
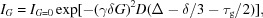
where *I_G_* is the observed signal intensity, *I*_*G*=0_ is the signal intensity in the absence of the gradient spin-echo, *G* is the gradient strength (Gauss cm^−1^), *D* is the diffusion coefficient (m^2^ s^−1^), γ is the gyromagnetic ratio of the observed nucleus, τ_g_ is the gradient recovery delay (s), Δ is the diffusion time (s) and δ is the duration of the gradient pulse (s).

## 3. Results and discussion   

**Figure 1:**
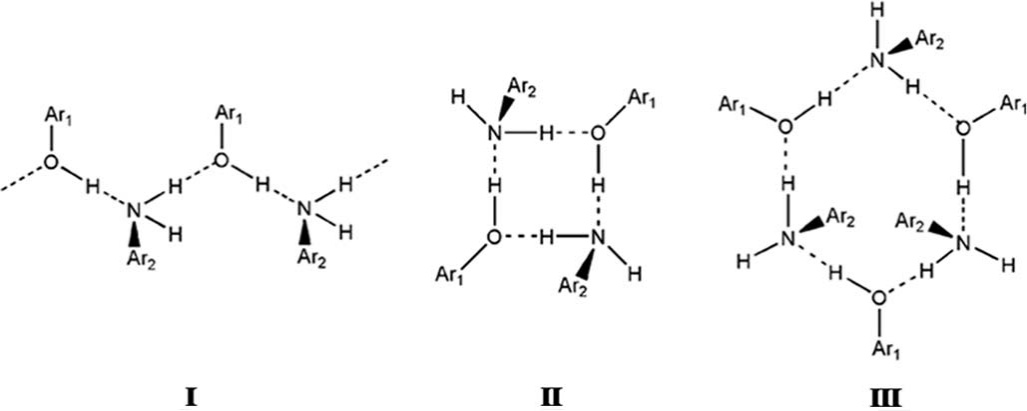
Supramolecular synthon possibilities in the aniline–phenol cocrystals in this study.

The supramolecular synthons referred to in this study are shown in Fig. 1 ▶. The main patterns are synthons **I** and **II**. Synthon **I**, which is an infinite open ⋯O—H⋯N—H⋯ chain, is the most common pattern in the family and is found in both 3- and 4-aminophenol prototypes. In 3-aminophenol, the second ‘free’ N—H group is involved in an N—H⋯π interaction. In 4-aminophenol, the infinite chains criss-cross each other to give closed hexamers, **III**, with a cooperative [⋯O—H⋯N—H⋯]_3_ arrangement. The main closed pattern in the family is the tetramer synthon **II**, [⋯O—H⋯N—H⋯]_2_, which is the target synthon in the present study.

### 3.1. Aniline–phenol recognition: general considerations   

A CSD study was performed to examine aniline–phenol recognition in aromatic compounds. A total of 176 hits were obtained that contain both aniline (—NH_2_) and phenol (—OH) fragments. Of this number, 77 (44%) contain O—H⋯N hydrogen bonds, the strongest hydrogen bond possible in this family. These 77 hits may be divided into 48 single-component and 29 multi-component structures. Analysis of the 48 single-component structures shows that 19 have the 3-aminophenol structure. Ten structures take the 4-aminophenol structure, which includes synthon **III**. Synthon **III** arises due to the compatibility between geometric and chemical factors of the —OH and —NH_2_ groups at the 1- and 4-positions of the aromatic ring. In the 3-aminophenol structure, the positional compatibility between —NH_2_ and —OH is lost, and geometrical factors play a major role in determining the final *herringbone* structure. As a result, the synthon pattern deviates from **III** and an additional N—H⋯π interaction is observed in this structure. The structure of 3-aminophenol is indicative of the fact that if the cooperativity between —NH_2_ and —OH groups is perturbed, the synthon pattern deviates from synthon **III**. Seven structures have the closed tetramer structure, **II**, while three have infinite ⋯O—H⋯N—H⋯ chains (synthon **I**) without N—H⋯π interactions. The remaining nine structures show interaction interference: absence of N—H⋯O [CSD refcodes AMNPHA (Haisa *et al.*, 1980 ▶), NODTIJ (Blagden *et al.*, 2001 ▶) and HIWNEH (Largeron *et al.*, 2008 ▶)]; finite chains [CSD refcodes GIVRIM (Mahmoud *et al.*, 1998 ▶), SADJAK (Kar *et al.*, 2010 ▶), UHEVOT (Bacchi *et al.*, 2009 ▶) and WURNHZ (Lu *et al.*, 2010 ▶)]; hexamer synthon composed of O—H⋯N, N—H⋯O, O—H⋯O, N—H⋯N [CSD refcodes PEJCAJ and PEJCAJ01 (Dey & Desiraju, 2006 ▶)].

**Figure 2:**
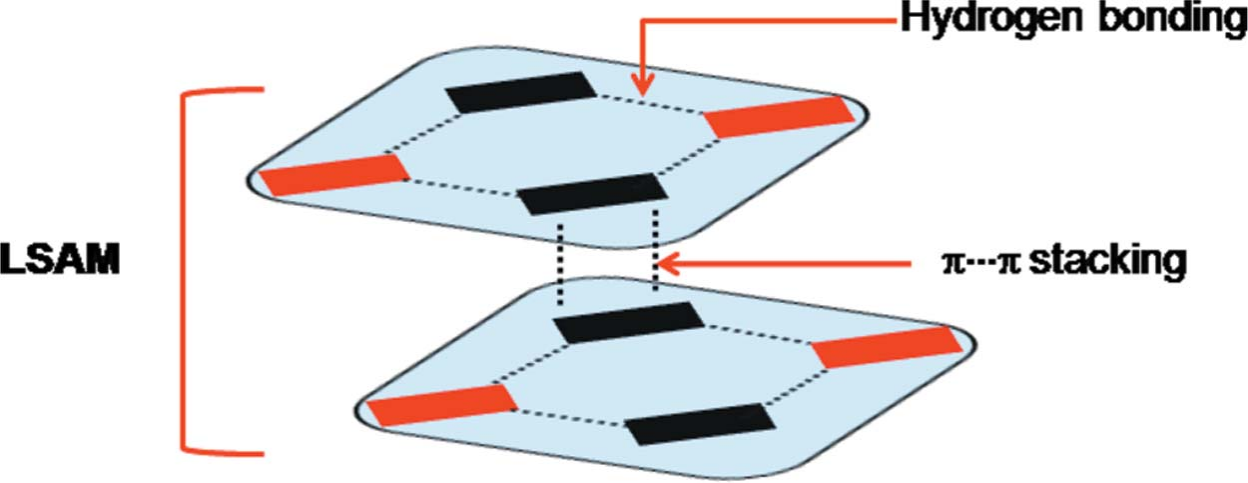
Construction of the target LSAM by amalgamation of hydrogen bonding and π⋯π stacking.

The 29 multi-component structures are distinct in that 14 of them are quite free from interference from other functionalities and show clear structural preferences. Ten of them have the [⋯O—H⋯N—H⋯]_3_ hexamer in the 4-aminophenol structure and three have the [⋯O—H⋯N—H⋯]_2_ tetramer (Van Bellingen *et al.*, 1971 ▶). The infinite ⋯O—H⋯N—H⋯ chain, without N—H⋯π interactions and without hexamers, is seen in one structure. Generally, the multi-component crystals take the 4-aminophenol structure; the tetramer is uncommon and other outlier structures are rarely seen. Could it be that the very formation of a multi-component crystal is already accompanied in the early stages with a funnelling into a certain pathway that is mediated by ⋯O—H⋯N—H⋯ hydrogen bonding? The following observations on the ten tetramer structures (seven single-component and three multi-component) are relevant: (i) in all three multi-component crystals [CSD refcodes FIDLIO, FIDLOU (Vangala *et al.*, 2004 ▶) and SARLEC (Loehlin *et al.*, 1998 ▶)], the aniline contains more than one —NH_2_ group and/or the phenol contains more than one —OH group; (ii) in five of the seven single-component crystals, π⋯π interactions (stacking of aromatic rings) are present. With these observations, we attempted to formulate a design strategy for aniline–phenol cocrystals that would lend themselves to NMR study in solution, in order that the presence of large synthons (LSAMs) might be detected in solution prior to crystallization. A synthon consisting of both hydrogen-bonded and π-stacked regions, as shown in Fig. 2 ▶, was identified as one such strategy.

### 3.2. Aniline–phenol recognition: crystal engineering   

#### 3.2.1. Design strategy for cocrystals   

The ability to anticipate packing patterns in crystal structures based on known structures is a daunting task when the database of known structures is small. It is even more difficult to design structures that are based on synthons which are not the most common ones in the respective family. CSD studies showed that tetramer structures are not the most common but that they may be favoured when there is an additional stability from π⋯π stacking (Hunter & Sanders, 1990 ▶). Given that we were searching for a synthon of the type shown in Fig. 2 ▶, our attention shifted naturally to synthon **II**. The design of aniline–phenol cocrystals based on synthon **II** is difficult because the aromatic rings can themselves be a part of a synthon (say with N—H⋯π) and interfere with other functionalities, notably the —NH_2_ group. Therefore, successful design of crystal structures containing synthon **II** may need steering groups which can form strong π⋯π interactions that may eventually decrease the interference from the rest of the molecule (Desiraju *et al.*, 2011 ▶). In this context, we chose 3,4,5-trichlorophenol, **1**, as the main compound in this study. The 2,3,4 isomer, **2**, was also used in some experiments. In **1**, the phenolic —OH group and the Cl atoms are well separated. The electron-deficient nature of the aromatic ring was also expected to favour π⋯π stacking. For steric and electronic reasons, phenol **1** was selected for cocrystallization with several halogenated anilines in order to obtain recurring packing patterns in the respective cocrystals. The coformers used are anilines **3**–**10**.

**Figure 3:**
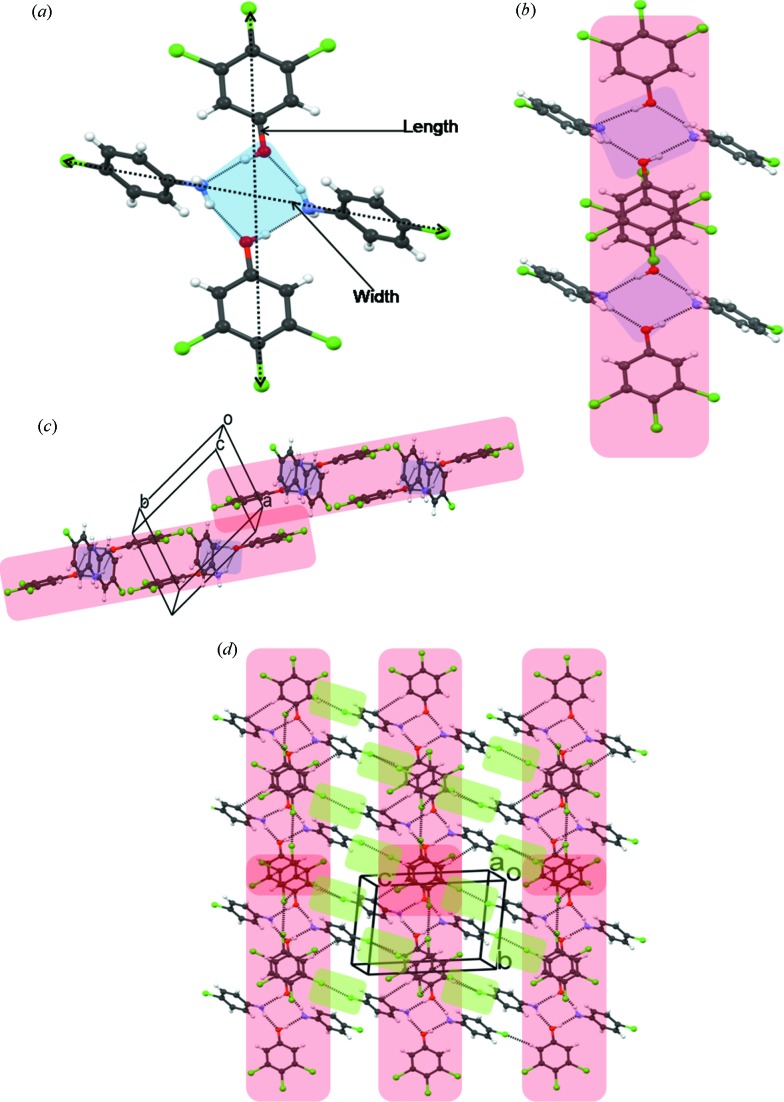
(*a*) Hydrogen-bonded tetramer synthon, **II**, in cocrystal **11**; (*b*) antiparallel π⋯π stacking of trichlorophenol rings in adjacent tetramers to give the LSAM (compare with Fig. 2 ▶); (*c*) arrangement of the LSAMs along [210]; (*d*) association of the LSAMs along [001] with halogen atom synthons (shown in green).

Phenol **1** was taken with 4-chloroaniline (**3**) in 1:1 *n*-hexane–MeOH in an equimolar ratio to give cocrystal **11** (Table 1 ▶). The primary synthon in this structure is the desired closed tetramer **II** (Fig. 3 ▶ *a*) which consists of alternating aniline and phenol molecules. The aniline ring is tilted at an angle of 53.4° to the phenol ring. The phenol rings in adjacent tetramers are stacked in an antiparallel manner roughly down the *a* axis (Fig. 3 ▶ *b*). The stacked dimer (octamer) in Fig. 3 ▶(*b*) is of great importance because it corresponds to the LSAM that is monitored with NMR in the second part of this study. The extended structure propagates in a sort of double layer that is aligned along [210] as shown in Fig. 3 ▶(*c*). The double layers are themselves loosely associated in the *c*-axis direction with halogen atom interactions (Fig. 3 ▶ *d*). The periphery of the double layer is halogen-rich.

Synthon **II** was expected to be of high modularity. The chloro-substitution pattern of the aniline may change the width of the module slightly but its length remains almost the same because it is a function of the molecular size of phenol **1**. This means that cocrystals that may be obtained when phenol **1** is taken with other related anilines should have nearly the same crystal structure as **11** with the *a* and *b* axes practically the same (within a particular crystal system or space group), and with the *c* axis varying slightly depending on the substitution pattern in the aniline. This expectation was borne out in practice (Table 1 ▶).

#### 3.2.2. Significance of LSAMs   

**Figure 4:**
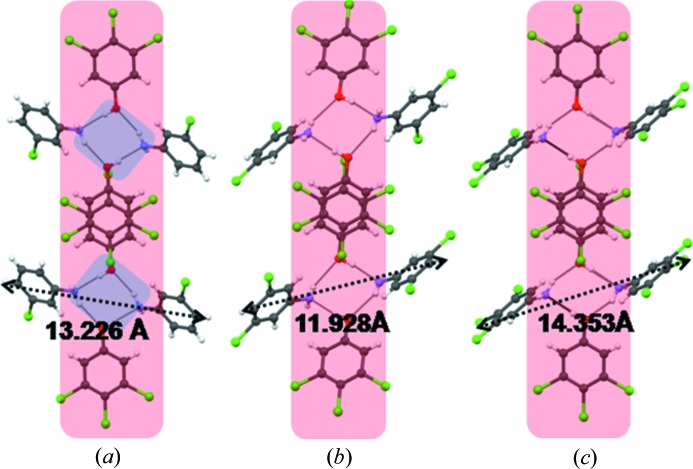
Octamer LSAMs in cocrystals **12** (*a*), **14** (*b*) and **15** (*c*). The width shown in these figures is calculated as the distances between the 4-substituents in two aniline molecules in the tetramer **II**. Compare with Fig. 3 ▶(*b*) and Fig. 2 ▶.

Crystal engineering seeks a modular way to describe a structure. Transfer of smaller structural units (synthons, LSAMs) simplifies the complex task of comparing interaction strengths to one of putting structural modules together. To this end, and analogous to the *Aufbau* modules proposed originally by Kitaigorodskii (1961 ▶), Ganguly & Desiraju (2010 ▶) proposed the concept of the LSAM. In this notation, a crystal can be dissected into long-range larger synthons which are modular and therefore the crystal structure can be described by different arrangements of these modules. Long-range synthons can be formed by various combinations of different small/large synthons. In the context of this paper, we consider O—H⋯N and N—H⋯O as the primary synthons, the tetramer synthon (**II**) as the larger secondary synthon and a combination of two tetramers through π⋯π interactions as the octamer LSAM. One of the objectives of this work is to find out how the LSAMs can be transferred from one structure to another and how this transferability contributes to the overall predictability of the crystal structures. The cocrystallization of **1** with **4**, **6** and **7** produced cocrystals **12**, **14** and **15**, respectively. As hypothesized in the preceding paragraph, all four structures adopt structures similar to that of **11** (Fig. 4 ▶).

**Figure 5:**
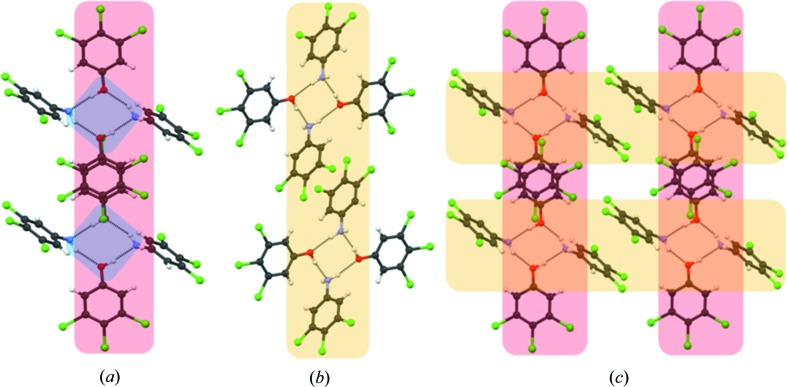
Cocrystal **15**. (*a*) LSAM formed by π⋯π stacking between molecules of 3,4,5-trichlorophenol, (*b*) alternative LSAM formed by π⋯π stacking between molecules of 3,4-dichloroaniline, (*c*) combination LSAM.

In cocrystal **12**, the change in position of the Cl atom on the aniline periphery causes a structural variation in the long direction. In **11**, Cl in the 4-position forms Cl⋯Cl halogen bonds with neighbouring LSAMs whereas in **12**, Cl in the 3-position forms an intra-LSAM halogen bond. Cocrystal **14** shows similarity with **12** in the organization along the long-axis dimension because the Cl-substituent positioning in **6** is similar to **4**. Curiously, this structure shows an unusually large excess electron density in the Fourier map in the 4-position of the aniline. This excess density may arise from a small number of domains of pure 3,4,5-trichlorophenol which have an O—H⋯O tetramer in its native structure. The implication is that tetramers of pure **1** and of the aniline–phenol adduct (**II**) are present in solution and that a small amount of the former is included in the cocrystal in a solid solution manner. Cocrystal **15** contains an aniline analogue, **7**, which is also substituted with Cl in 3- and 4-positions. The Cl in the 3-position shows partial occupancy and, therefore, **7** behaves like a 3,4,5-trichloroaniline. This substitution pattern favours antiparallel stacking (Fig. 5 ▶). Therefore, in **15**, there is stacking of both phenol and aniline rings. A very short Cl⋯Cl type I contact of length 3.078 (1) Å is observed which connects two LSAMs.

#### 3.2.3. LSAM organization: tuning with halogen bonds   

**Figure 6:**
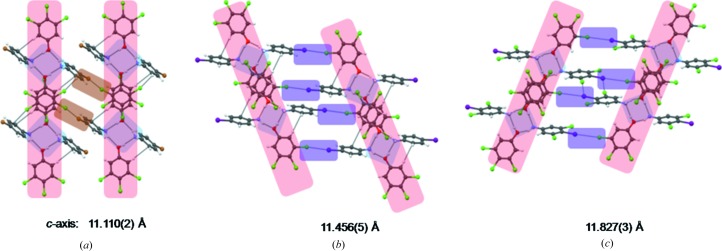
Halogen bonds used in tuning the longer direction in the cocrystals (*a*) **16**, (*b*) **17** and (*c*) **18**.

The structure of the octamer LSAM in cocrystal **11** indicates that Cl in the 4-position in **3**, which lies on the periphery of the LSAM, controls the organization of the LSAMs in the direction of the long axis. In this context, it was assumed that replacing Cl with Br or I may lead to better control in the long-axis direction. Accordingly, cocrystals **16** and **17** were prepared. When **1** is cocrystallized with **8**, it gives **16** (Table 1 ▶). The LSAM remains intact as reflected in the *a-* and *b* directions. The *c* direction (which is longer than the corresponding length in **11**) is controlled by a Br⋯Cl type II interaction which is 3.489 (1) Å in length. With the success of the hypothesis that the halogen bond can control the strength and directionality in the long crystal axis direction, we tried to cocrystallize **9** with **1** in the next step, resulting in cocrystal **17**. The replacement of Br with I results in a cocrystal with a similar structure (Table 1 ▶). The *c* direction is determined by an I⋯Cl interaction of 3.583 (1) Å in length. When **1** is crystallized with **10** it results in **18**, which has a longer *c* axis than **15** (Table 1 ▶). These results show that the increased control in engineering the packing in the longer crystal axis direction (mostly the *c* axis in our study) can be obtained by introducing halogen bonds (Metrangolo *et al.*, 2005 ▶) oriented in that direction (Fig. 6 ▶). It is also shown that if insulating interactions with variable strengths are incorporated in almost perpendicular directions, it is possible to predict the crystal structures. The notable point in these structures is that the common structural part is not restricted to tetramer synthon **II**. The larger octamer LSAM, which is obtained by π⋯π stacking of two tetramers, is impressively repeated in as many as seven cocrystal structures. This observation leads to the possibility that LSAMs may also exist in solution.

#### 3.2.4. Transferability of tetramer and LSAM   

**Figure 7:**
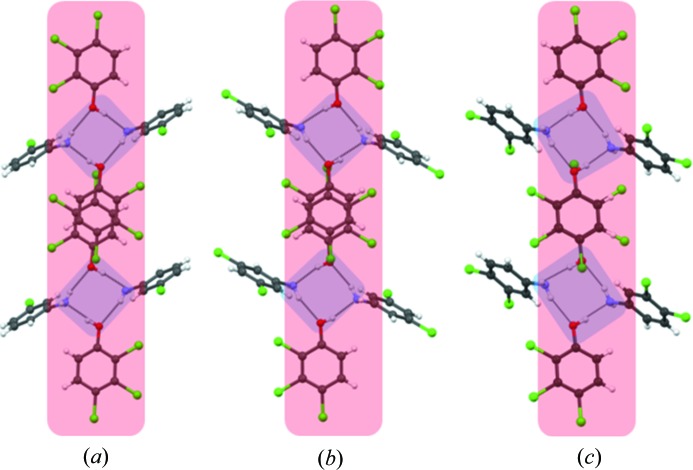
Cocrystal structures formed with **2** and showing the presence of the octamer LSAM: (*a*) **20**, (*b*) **21**, (*c*) **22**.

The octamer LSAM remains intact even when phenol **2** is cocrystallized with **3**, **6** and **7** to produce **20**, **21** and **22** (Fig. 7 ▶). The tetramer **II** is translated to LSAMs with an inversion centre between two molecules of **2** to facilitate the stacking. The increase in the *c*-axis length in **21** compared with **20** (Table 1 ▶) results from the symmetric arrangement of Cl atoms in **6**, which gives rise to an extra Cl⋯Cl type I contact, compatible with triclinic inversion symmetry. It is important to note that the same LSAM is found in cocrystals formed by phenols **1** and **2**, as manifested in the lengths of the two shorter cell axes in cocrystals **20**, **21** and **22** being practically the same as the corresponding ones in cocrystals **11**, **12** and **14**–**18**.

**Figure 8:**
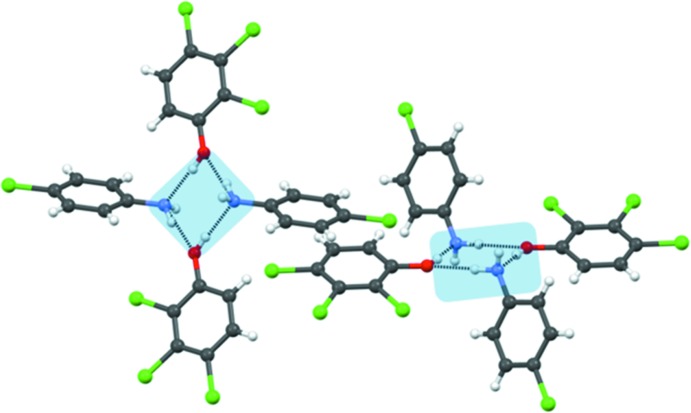
Tetramer synthons observed in cocrystal **19.** The LSAM is not observed.

Cocrystal **19**, on the other hand, shows the presence of tetramer **II** but the LSAM is not the same (Fig. 8 ▶). Instead of phenol–phenol antiparallel stacking as in **20**, **21** and **22**, there is phenol–aniline stacking between molecules of **2** and **4**. Although only four cocrystals of phenol **2** were studied, it appears that there is more structural variability in these structures compared with the seven cocrystals formed by phenol **1**. Perhaps the more distant positioning of the —OH group and Cl atoms in **1** leads to a certain amount of insulation and consequent predictability of the crystal structures.

#### 3.2.5. Distorted LSAMs   

**Figure 9:**
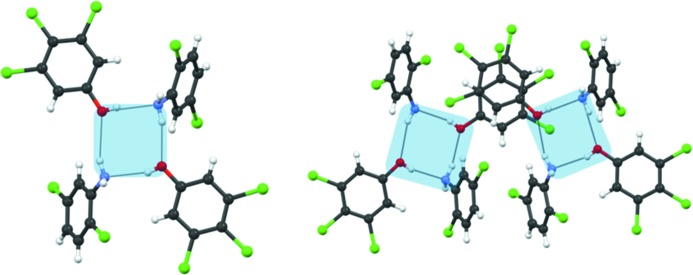
Distorted LSAM observed in cocrystal **13**.

The LSAM is a finite entity and its size and shape depends on the positioning of the functional groups. When **1** is cocrystallized with **5** it gives **13**, which contains distorted LSAMs (Fig. 9 ▶). The uneven positioning of Cl atoms on the ring periphery of 2,5-dichloroaniline restricts the structure from adopting the common LSAM observed in cocrystals **11**, **12**, **14**, **15**, **16**, **17** and **18**. Two 3,4,5-trichloro­phenol molecules are still stacked with each other by π⋯π interactions, but unlike the other structures, they are not stacked in an antiparallel fashion. Therefore, the distorted LSAM in this structure may be attributed to the uneven positioning of Cl atoms on the aniline periphery, which facilitates the formation of C—H⋯Cl hydrogen bonds. Perhaps this hints that hydrogen bonding precedes stacking in solution.

#### 3.2.6. Transferability of the tetramer synthon to other cocrystals   

**Figure 10:**
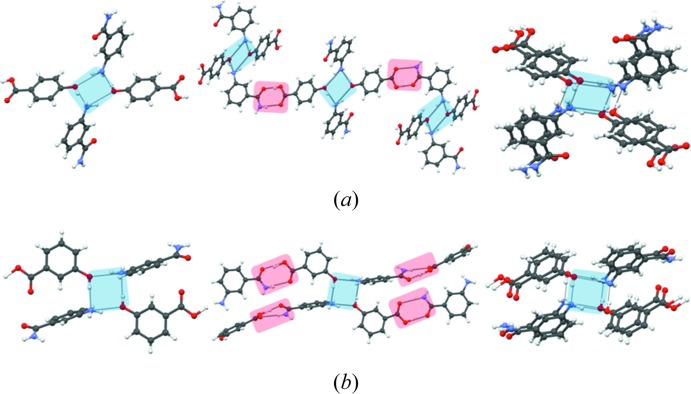
Transferability of the tetrameric synthon to other cocrystals: (*a*) **23**, (*b*) **24**.

The next objective of the study was to test the applicability of the proposed design strategy: the transferability of the tetrameric synthons was checked in more complex multi-functional non-halogenated cocrystals where other hydrogen bonding is possible. In other words, what is the robustness of synthon **II** in the presence of other strong hydrogen bonds? The coformers used in these studies contain functional groups like amides and acids which are able to form strong hydrogen bonds. The observation of the tetrameric synthon in these cocrystals would depend upon the relative positions of the functional groups as they are not completely insulated from each other. It is well known that functional groups in 1- and 4- positions often interfere, especially if both of them are quite strong and directional in nature. Keeping this aspect of crystal design in mind, we chose 3-aminobenzamide for crystallization with 4-hydroxybenzoic acid and 3-aminobenzoic acid. When 3-aminobenzamide was cocrystallized with 4-hydroxybenzoic acid, it resulted in the formation of a 1:1 cocrystal (**23**) which shows the presence of a tetramer synthon (Fig. 10 ▶). The cocrystallization of 3-aminobenzamide with 3-aminobenzoic acid gives cocrystal **24** which also sustains tetramer **II** (Fig. 10 ▶).

**Figure 11:**
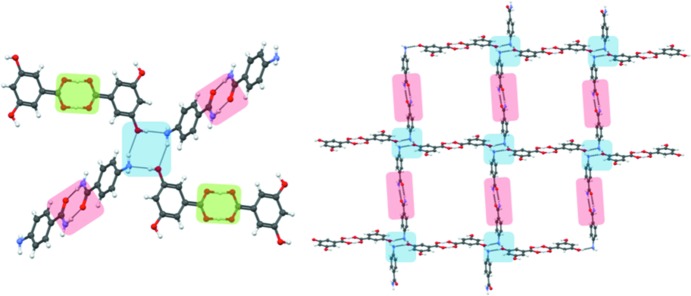
The cocrystal of 3,5-dihydroxybenzoic acid and 4-aminobenzamide (**25**) shows the formation of the tetrameric synthon. Thereafter, it translates into the formation of a predictable network.

When 4-aminobenzamide is cocrystallized with 3,5-dihydroxybenzoic acid, it results in cocrystal **25**, which is also sustained by the aniline–phenol tetramer **II** (Fig. 11 ▶). The interesting aspect in this structure is the presence of amide–amide and acid–acid interactions. This is very rare in the sense that when acid and amide are present in a cocrystal system they usually tend to form acid–amide heterosynthons *in lieu* of homosynthons (Allen *et al.*, 1999 ▶). A CSD search performed on the aromatic acid–aromatic amide multi-component crystals gave 37 hits, among which 13 (35.1%) structures have amide–amide dimers. A manual analysis of these 13 structures revealed that there is no structure wherein the acid–acid dimer is also present. This implies that aniline–phenol recognition is so persistent that even the ‘normal’ behaviour of acid and amide functionalities is modified. This observation also reinforces the strength and robustness of the tetramer synthon.

### 3.3. Synthon structure in solution   

The understanding of crystal nucleation and growth is still at a nascent stage (Davey *et al.*, 2013 ▶; Erdemir *et al.*, 2009 ▶; Weissbuch *et al.*, 2003 ▶; Derdour & Skliar, 2012 ▶; Vekilov, 2010 ▶). The region of the crystallization reaction coordinate (structural landscape) between the late stages of nucleation and the early stages of growth is still far from understood (Desiraju, 2007 ▶). In this context, it is of interest to know whether synthons that have been identified in crystals may actually be defined in solution. There are very few studies available to this end. An FTIR study by Davey and co-workers of tetrolic acid shows the presence of dimer and catemer synthons in solution (Parveen *et al.*, 2005 ▶). In a more recent study, ter Horst and co-workers performed an FTIR and Raman study on isonicotin­amide to probe the formation of homo- and heterosynthons in solution (Kulkarni *et al.*, 2012 ▶). Both of these studies indicate that there is a possibility of carry-over of the small synthons from solution to the crystal if classical nucleation theory operates. However, these studies are silent about larger synthons and LSAMs. In the present study, the robustness of the tetramer synthon **II** and corresponding octamer LSAM prompted us to look at the aggregation behaviour in solution, through the following NMR studies.

#### 3.3.1. Chemical shift perturbation as a function of concentration   

**Figure 12:**
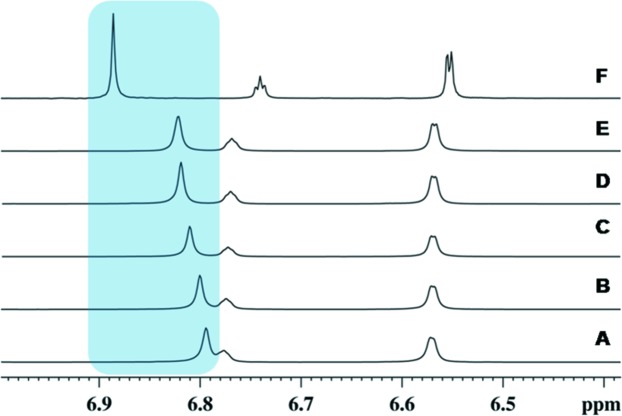
Chemical shift as a function of dilution (**A**→**F**) of **14** in CDCl_3_. In **F**, δ 6.88 (H2, H6 of **1**), δ 6.74 (H4 of **6**), δ 6.55 (H2, H6 of **6**). A significant upfield shift of the H2, H6 protons of **1**, combined with overall line-narrowing is observed upon dilution from **E** to **F**.

Fig. 12 ▶ shows one-dimensional ^1^H NMR spectra of **14** in CDCl_3_ at various dilutions. A single set of resonances that can be assigned to the aniline and phenol fragments is observed. This indicates that in solution the sample is homogeneous and that the fragments do not exhibit conformational exchange. A downfield shift of the 2, 6 protons (δ ∼ 6.8 p.p.m.) of **1** as a function of dilution (**A**→**E**) is clearly observed, indicating stacking of the aromatic rings. The abrupt shift in going from **E** to **F** shows that aromatic stacking interactions are lost at this point. The aromatic protons of aniline (δ ∼ 6.55 and ∼ 6.74 p.p.m.) are largely unaffected upon dilution. Noting that the phenol rings are stacked in the crystal structure of **14**, the downfield shift of the phenol protons may arise from the presence of the octamer species in solution, or it may arise from a simple stacking of isolated phenol molecules. To distinguish between these possibilities, samples **A**, **E** and **F** were studied to estimate association and comparative molecular sizes using NOEs, measurement of *T*_1_ relaxation rates and translational diffusion coefficients.

#### 3.3.2. One-dimensional difference NOE experiments   

**Figure 13:**
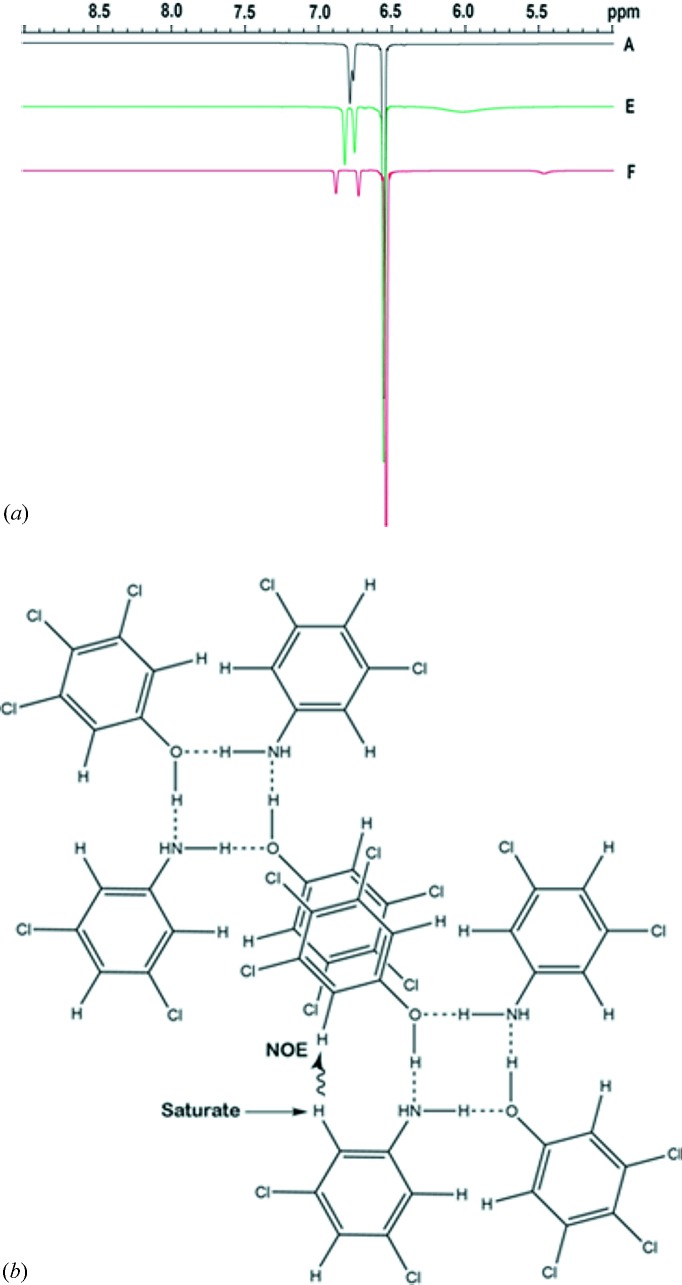
(*a*) NOE difference spectra of solutions **A**, **E** and **F**. Negative NOE peaks are observed in the difference spectra at all three dilutions. The difference NOE peaks indicate that the hydrogen bonding between **1** and **6** is intact. (*b*) The saturation of the H2, H6 protons of **6** should result in a NOE effect on the H2, H6 protons of **1**.

Having established the aromatic stacking interactions in solution, we proceeded to an NOE (Neuhaus & Williamson, 2000 ▶) study of **14**, to examine the possibility of aniline–phenol hydrogen bonding in solution, and in turn the presence of tetramer **II**. Saturation of the H2, H6 protons of **6** and the resulting NOE on the proximal H2, H6 protons of **1** (Fig. 13 ▶ *a*) confirms hydrogen bonding between —NH_2_ and —OH groups. The peaks at δ 6.7 and 6.9 p.p.m. are the NOE difference peaks of the H2, H6 protons of **1**. The presence of these peaks at all three dilutions indicate that the hydrogen bonding between **1** and **6** is intact in solution. Based on the robustness of tetramer synthon **II** in the crystal structures in this study, the CSD results on earlier aniline–phenol cocrystals, and the CSP results given by Dey *et al.* (2005 ▶), we conclude that the hydrogen bonding is prevalent in a cyclic closed tetramer, **II**, in solution (Fig. 13 ▶ *b*). Finally these data, when taken in conjunction with the chemical shift data discussed above, would indicate that the stacking interactions responsible for the formation of the LSAM is *preceded* by the hydrogen-bonded association of **1** and **6** to form the tetramer synthon.

#### 3.3.3. Longitudinal relaxation time constants (*T*_1_)   

^13^C *T*_1_ relaxation time constants (Table 2 ▶) were measured using the *T*_1_ inversion recovery pulsed method (Claridge, 2008 ▶). In general, for solutes in non-viscous solvents, the *T*_1_ relaxation time constant increases as the molecular size decreases. This is due to a decrease in the rotational correlation time. From the values in Table 2 ▶, it is clear that *T*_1_ increases from **A**→**E**→**F**, in other words with increasing dilution; this is strongly suggestive of dissociation from octamer to tetramer species.

#### 3.3.4. Estimation of translational diffusion coefficients (*D*_s_)   

To differentiate the sizes of various molecular aggregates in solution, the translational diffusion coefficients of molecular species in solutions **A**, **E** and **F** were measured using the bipolar pulsed gradient stimulated echo sequence (Stejskal & Tanner, 1965 ▶; Johnson, 1999 ▶). Fig. S4 in the supporting information is an example of the one-dimensional diffusion NMR spectra obtained at 600 MHz. Fig. S5 shows the two-dimensional representation of the experimentally derived translational diffusion coefficients. Since the translational diffusion coefficient is inversely proportional to the radius of the molecule that is diffusing, a decrease in radius should result in an increase in *D*_s_. This is exactly what is observed in going from **A**→**E**→**F** (Table 3 ▶).

#### 3.3.5. 1,2,3-Trichlorobenzene: a control compound to identify stacking in the LSAM   

**Figure 14:**
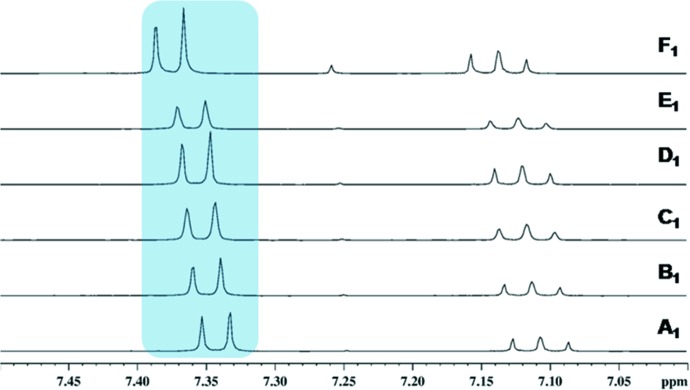
^1^H chemical shifts in dilution experiments (**A_1_**→**F_1_**) for 1,2,3-trichloro­benzene, **26**, in CDCl_3_.

1,2,3-Trichlorobenzene, **26**, was chosen as a test compound to probe changes in chemical shifts that can arise from the stacking of the 1,2,3-trichlorophenyl rings. In the crystal, **26** exists as a two-molecule antiparallel stack (see supporting information) (Hazell *et al.*, 1972 ▶). It was anticipated that this antiparallel stack would persist in solution but obviously there would be no hydrogen bonding as seen in cocrystal **14**. The effect of concentration on this stacking interaction was studied in a manner similar to that for **14**. Fig. 14 ▶ shows one-dimensional ^1^H NMR spectra of **26** at different dilutions. The molar concentration of **26** in each sample was identical to that of **1** and **6** in each of the corresponding samples (**A**→**F**) of **14**. The abrupt change in chemical shift in going from **E**→**F** once again indicates that the stacking interaction in lost upon dilution, at a definite point. The translational diffusion coefficients measured for **26** (Table 4 ▶) indicate no change at dilutions **A_1_** and **E_1_** and a small change in **F_1_**, in accordance with the above interpretation.

The measured values of *T*_1_ and *D*_s_ for **26** in samples **A_1_**, **E_1_** and **F_1_** are given in Table 4 ▶. The two-dimensional DOSY plots for **A_1_**, **E_1_** and **F_1_** are shown in the supporting information (§S4). The larger values of *T*_1_ in the case of **26** strongly suggest that the aggregates in **A_1_** and **E_1_** are smaller than the corresponding aggregates in **A** and **E** of **14**, as would be expected. The size of the dimer of **26** is roughly four times smaller than the octamer LSAM derived from **14**. Additional evidence for this interpretation comes from the values of the measured translational diffusion coefficients. From the values given in Table 4 ▶, it is clear that the species in **A_1_** and **E_1_** are significantly smaller in size than those in **A** and **E**, although it is not possible to state that it is exactly four times smaller. As mentioned above, crystallographic studies have established that 1,2,3-trichlorobenzene forms a stacked antiparallel dimer in the solid state. Thus, it may be concluded that **A_1_** and **E_1_** represent the stacked antiparallel dimers of 1,2,3-trichloro­benzene, and **F_1_** the monomer form. In summary, the solution NMR studies indicate that in the phenol–aniline system investigated here, the LSAMs are assembled *via* stacking interactions of tetramer synthons. The sequence of events is therefore established: hydrogen bonding between aniline and phenol comes first and π⋯π stacking follows.

One-dimensional ^15^N spectra (supporting information) recorded on samples **A**, **E** and **F** show no change in the chemical shift positions as a function of concentration. Since ^15^N chemical shifts are very sensitive to the environment, this observation reinforces the conclusion that hydrogen bonding persists in **F**. The absence of other peaks in the ^15^N spectrum of **F** further points towards the presence of a single species within the limits of detection.

#### 3.3.6. Presence of a hydrogen-bonded tetramer in solution   

The NMR experiments point to a hydrogen-bonded and stacked aggregate at higher concentrations that loses the stacking interactions upon dilution to give a hydrogen-bonded aggregate. This indicates that hydrogen bonding in this system is stronger than stacking. We have interpreted these results in terms of a hydrogen-bonded tetramer which is stacked to form dimers, trimers and in the limit of high concentrations *n*-mers of tetramers. It is desirable to rule out a trivial scenario in which the highest concentration species is a stacked structure of hydrogen-bonded dimers. The evidence for this is as follows: *T*_1_ values in **A** through to **E** indicate that the species size is gradually decreasing. This is corroborated by the diffusion coefficients and is in accord with a stacked *n*-mer in **A** reducing to a stacked dimer in **E**. It is highly unlikely that the stacked *n*-mers are constituted with hydrogen-bonded dimers or even trimers because hydrogen bonding is a stronger interaction than stacking. The tetramer structure, which is ‘saturated’ with respect to hydrogen-bonded capability, is a far more likely candidate to be the basic synthon in the structure. Circumstantial evidence for this is also provided by the distorted LSAM in **13**, which is a high-*Z*′ crystal structure where stacking is distorted but where the hydrogen-bonded tetramer is preserved fully.

## 4. Conclusions   

The rational synthesis of cocrystals is a difficult task. It becomes even more difficult when there are multiple synthon possibilities in the system and one tries to design a cocrystal wherein the desired synthon is not the most probable one. In this study, a number of aniline–phenol cocrystals have been isolated and shown to contain a large but robust octamer synthon, or LSAM, stabilized by cooperative N—H⋯O—H⋯ hydrogen bonding and π⋯π stacking, and where the hydrogen bonding defines a closed tetramer. It is noteworthy that a synthon this large and complex is repeated in so many crystal structures. NMR experiments in solution demonstrate conclusively the presence of hydrogen bonding and stacking interactions in solution, the likely presence of a tetramer and octamer and the preferred aggregation *via* hydrogen bonding as compared with stacking. The NMR studies are a way of establishing the hierarchy in which the various intermolecular interactions are established in the molecular association process. This could be a reason why the tetramers are transferrable even to cocrystals that are dominated by strong hydrogen bonds (**23**, **24**, **25**). To our knowledge, this is the first time that such stepwise formation of intermolecular interactions has been monitored. The LSAM is, in a true sense, an additive representation of supramolecular synthons because the geometrical and chemical information implied in supramolecular synthons add up to make a LSAM, which is significant in the very late stages of nucleation. Therefore, it is reasonable to assume that LSAMs can be more useful than individual supramolecular synthons in crystal design, *especially when the finer details like unit-cell dimensions are sought to be engineered*. The observation of a LSAM in solution and its use in crystal engineering in a predictable way may therefore pave the way for control of crystal packing with greater predictability and precision.

## Supplementary Material

Crystal structure: contains datablock(s) global, 11, 12, 13, 14, 15, 16, 17, 18, 19, 20, 21, 22, 23, 24, 25. DOI: 10.1107/S2052252514012081/bi5032sup1.cif

Details of synthesis/crystallization, CSD searches, further crystallographic information, NMR details. DOI: 10.1107/S2052252514012081/bi5032sup2.pdf

Structure factors: contains datablock(s) 11. DOI: 10.1107/S2052252514012081/bi503211sup4.fcf

Structure factors: contains datablock(s) 12. DOI: 10.1107/S2052252514012081/bi503212sup5.fcf

Structure factors: contains datablock(s) 13. DOI: 10.1107/S2052252514012081/bi503213sup6.fcf

Structure factors: contains datablock(s) 14. DOI: 10.1107/S2052252514012081/bi503214sup7.fcf

Structure factors: contains datablock(s) 15. DOI: 10.1107/S2052252514012081/bi503215sup8.fcf

Structure factors: contains datablock(s) 16. DOI: 10.1107/S2052252514012081/bi503216sup9.fcf

Structure factors: contains datablock(s) 17. DOI: 10.1107/S2052252514012081/bi503217sup10.fcf

Structure factors: contains datablock(s) 18. DOI: 10.1107/S2052252514012081/bi503218sup11.fcf

Structure factors: contains datablock(s) 19. DOI: 10.1107/S2052252514012081/bi503219sup12.fcf

Structure factors: contains datablock(s) 20. DOI: 10.1107/S2052252514012081/bi503220sup13.fcf

Structure factors: contains datablock(s) 21. DOI: 10.1107/S2052252514012081/bi503221sup14.fcf

Structure factors: contains datablock(s) 22. DOI: 10.1107/S2052252514012081/bi503222sup15.fcf

Structure factors: contains datablock(s) 23. DOI: 10.1107/S2052252514012081/bi503223sup16.fcf

Structure factors: contains datablock(s) 24. DOI: 10.1107/S2052252514012081/bi503224sup17.fcf

Structure factors: contains datablock(s) 25. DOI: 10.1107/S2052252514012081/bi503225sup18.fcf

CCDC references: 962085, 962086, 962087, 962088, 962089, 962090, 962091, 962092, 962094, 962093, 962095, 967791, 962096, 962097, 962098

## Figures and Tables

**Table:** 

	**11**	**12**	**13**	**14**	**15**
Formula	C_6_H_3_Cl_3_O·C_6_H_6_ClN	C_6_H_3_Cl_3_O·C_6_H_6_ClN	C_6_H_3_Cl_3_O·C_6_H_5_Cl_2_N	C_6_H_3_Cl_3_O·C_6_H_5_Cl_2_N	C_6_H_3_Cl_3_O·C_6_H_5_Cl_2_N
Crystal system	Triclinic	Monoclinic	Monoclinic	Monoclinic	Triclinic
Space group	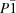	*P*2_1_/*c*	*P*2_1_	*I*2/*a*	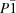
*a* (Å)	7.0243 (14)	6.9676 (7)	7.0572 (6)	22.638 (5)	7.0681 (6)
*b* (Å)	9.4152 (18)	21.336 (2)	15.4665 (13)	7.2553 (11)	9.5008 (8)
*c* (Å)	10.928 (2)	9.1861 (10)	13.2112 (11)	18.013 (3)	11.4095 (9)
α (°)	82.750 (6)	90	90	90	85.402 (6)
β (°)	79.147 (6)	99.139 (7)	98.980 (7)	90.767 (9)	83.071 (6)
γ (°)	76.703 (5)	90	90	90	71.211 (5)
Volume (Å^3^)	688.2 (2)	1348.3 (2)	1424.3 (2)	2958.3 (9)	719.36 (11)
*Z*	2	4	4	8	2
CCDC No.	962085	962086	962087	962088	962089

**Table:** 

	**16**	**17**	**18**	**19**	**20** †
Formula	C_6_H_3_Cl_3_O·C_6_H_6_BrN	C_6_H_3_Cl_3_O·C_6_H_6_IN	C_6_H_4_ClIN·C_6_H_3_Cl_3_O	C_6_H_3_Cl_3_O·C_6_H_6_ClN	C_6_H_3_Cl_3_O·C_6_H_6_ClN
Crystal system	Triclinic	Triclinic	Triclinic	Monoclinic	Triclinic
Space group	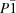	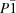	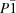	*P*2_1_/*c*	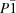
*a* (Å)	7.0562 (15)	7.083 (3)	7.107 (2)	7.851 (5)	7.208 (9)
*b* (Å)	9.373 (2)	9.354 (4)	9.498 (3)	11.865 (7)	9.333 (10)
*c* (Å)	11.110 (2)	11.456 (5)	11.827 (3)	14.891 (8)	10.884 (13)
α (°)	83.358 (6)	84.118 (7)	85.425 (6)	90	99.035 (14)
β (°)	79.173 (6)	79.555 (7)	81.804 (6)	106.79 (3)	107.107 (6)
γ (°)	76.588 (5)	76.553 (7)	71.851 (5)	90	102.219 (10)
Volume (Å^3^)	700.0 (2)	724.5 (5)	750.4 (4)	1328.0 (14)	664.8 (14)
*Z*	2	2	2	4	2
CCDC No.	962090	962091	962092	962094	962093

**Table:** 

	**21**	**22** †	**23**	**24**	**25**
Formula	C_6_H_3_Cl_3_O·C_6_H_5_Cl_2_N	C_6_H_3_Cl_3_O·C_6_H_5_Cl_2_N	C_7_H_6_O_3_·C_7_H_8_N_2_O	C_7_H_6_O_3_·C_7_H_8_N_2_O	C_14_H_12_O_8_·2C_7_H_8_N_2_O
Crystal system	Triclinic	Triclinic	Monoclinic	Monoclinic	Triclinic
Space group	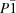	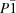	*C*2/*c*	*P*2_1_/*c*	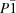
*a* (Å)	7.1441 (8)	7.2060 (8)	24.698 (2)	12.410 (15)	4.760 (2)
*b* (Å)	9.3027 (10)	9.2558 (10)	5.1072 (5)	5.124 (6)	11.501 (6)
*c* (Å)	11.8726 (13)	11.3203 (12)	20.6682 (19)	20.06 (2)	12.539 (6)
α (°)	77.966 (5)	99.693 (7)	90	90	77.081 (6)
β (°)	74.889 (5)	99.616 (7)	99.673 (12)	92.901 (14)	86.975 (6)
γ (°)	77.979 (5)	101.387 (7)	90	90	81.302 (6)
Volume (Å^3^)	735.06 (14)	713.76 (14)	2570.0 (4)	1274 (2)	661.3 (5)
*Z*	2	2	8	4	1
CCDC No.	962095	967791	962096	962097	962098

† Structures **20** and **22** are reported using their formal reduced cells, which have obtuse rather than acute angles. The cells are nonetheless comparable with those of **11**, **15**, **16**, **17**, **18** and **21**; all of these cocrystals are essentially isostructural.

**Table 2:** *T*_1_ relaxation time constants (s) for solutions **A**, **E** and **F**

	3,4,5-Trichlorophenol	3,5-Dichloroaniline	
	C2, C6	C2, C6	C4
**A**	3.60	2.15	3.05
**E**	4.31	2.77	3.58
**F**	5.64	3.42	4.85

**Table 3:** Translational diffusion coefficients (*D*_s_ × 10^−10^ m^2^ s^−1^) measured for **14**

Sample	Translational diffusion coefficient†
**A**	12.63 ± 0.21
**E**	16.19 ± 0.11
**F**	20.69 ± 0.25

† *D*_s_ values are reported for experiments carried out with a diffusion delay period of 100 ms.

**Table 4:** Measured *T*_1_ (s) and *D*_s_ (×10^−10^ m^2^ s^−1^) values for 1,2,3-trichlorobenzene, **26**

	*T*_1_		*D*_s_ †
	C5	C4, C6	
**A_1_**	6.16	5.90	20.51 ± 0.25
**E_1_**	6.44	6.81	20.59 ± 0.14
**F_1_**	5.68	5.83	23.47 ± 0.17

† *D*_s_ values are reported for experiments carried out with a diffusion delay period of 100 ms.
