# Supplementary material for: Aniline–phenol recognition: from solution through supramolecular synthons to cocrystals
Source: IUCrJ. 2014 Jun 12;1(Pt 4):228–39. doi: 10.1107/S2052252514012081 (PMC4107923; doi:10.1107/S2052252514012081)

IUCrJ

Volume 1 (2014)

Supporting information for article:

Aniline–phenol recognition: from solution through supramolecular synthons to cocrystals

Arijit Mukherjee, Karuna Dixit, Siddhartha P. Sarma and Gautam R. Desiraju

S1. Crystallization details

3,4,5-trichlorophenol: 4-chloroaniline (**11**): The two compounds were taken together in a mortar in 1:1 ratio and ground with a few drops of MeOH. The grinding process was repeated thrice. Solutions of resulting powder were kept for crystallizations. Diffraction quality crystals were obtained from a 1:1 solvent mixture of *n*-hexane and MeOH after 3-4 days by solvent evaporation.

3,4,5-trichlorophenol: 3-chloroaniline (**12**): The phenol was taken in a mortar and a few drops of 3-chloroaniline were added to it. The mixture was ground and the grinding process was repeated thrice. Solutions of resulting powder were kept for crystallizations. Diffraction quality crystals were obtained from $CHCl_3$ after 3-4 days by solvent evaporation.

3,4,5-trichlorophenol: 2,5-dichloroaniline (**13**): The two compounds were taken together in a mortar in 1:1 ratio and ground with a few drops of MeOH. The grinding process was repeated thrice. Solutions of resulting powder were kept for crystallizations. Diffraction quality crystals were obtained from $CHCl_3$ after 3-4 days by solvent evaporation.

3,4,5-trichlorophenol:3,5 –dichloroaniline (**14**): The two compounds were taken together in a mortar in 1:1 ratio and ground with a few drops of MeOH. The grinding process was repeated thrice. Solutions of resulting powder were kept for crystallizations. Diffraction quality crystals were obtained from acetone after 3-4 days by solvent evaporation.

3,4,5-trichlorophenol: 3,4-dichloroaniline (**15**): The two compounds were taken together in a mortar in 1:1 ratio and ground with a few drops of MeOH. The grinding process was repeated thrice. Solutions of resulting powder were kept for crystallizations. Diffraction quality crystals were obtained from CH_2Cl_2 after 3-4 days by solvent evaporation.

3,4,5-trichlorophenol:4-bromoaniline (**16**): The two compounds were taken together in a mortar in 1:1 ratio and ground with a few drops of MeOH. The grinding process was repeated thrice. Solutions of resulting powder were kept for crystallizations. Diffraction quality crystals were obtained from acetone after 3-4 days by solvent evaporation.

3,4,5-trichlorophenol: 4-iodoaniline (**17**): The two compounds were taken together in a mortar in 1:1 ratio and ground with a few drops of MeOH. The grinding process was repeated for thrice. Solutions of resulting powder were kept for crystallizations. Diffraction quality crystals were

obtained from a 1:1 solvent mixture of *n*-hexane and MeOH after 3-4 days by solvent evaporation.

3,4,5-trichlorophenol: 3-chloro-4-iodoaniline (**18**): The two compounds were taken together in a mortar in 1:1 ratio and ground with a few drops of MeOH. The grinding process was repeated thrice. Solutions of resulting powder were kept for crystallizations. Diffraction quality crystals were obtained from MeOH after 3-4 days by solvent evaporation.

2,3,4-trichlorophenol: 4-chloroaniline (**19**): The two compounds were taken together in a mortar in 1:1 ratio and ground with a few drops of MeOH. The grinding process was repeated thrice. The resulting powder was put for sublimation. Needle like crystals were obtained after two days.

2,3,4-trichlorophenol: 3-chloroaniline (**20**):The phenol was taken in a mortar and a few drops of 3-chloroaniline were added to it. The mixture was ground and the grinding process was repeated thrice. The resulting powder was put for sublimation. Needle like crystals were obtained after two days.

2,3,4-trichlorophenol: 3,5-dichloroaniline (**21**): The two compounds were taken together in a mortar in 1:1 ratio and ground with a few drops of MeOH. The grinding process was repeated thrice. The resulting powder was dissolved in different solvents. Nice crystals were obtained from EtOH after 3-4 days by solvent evaporation.

2,3,4-trichlorophenol: 3,4-dichloroaniline (**22**): The two compounds were taken together in a mortar in 1:1 ratio and ground with a few drops of MeOH. The grinding process was repeated thrice. The resulting powder was put for sublimation. Needle like crystals were obtained after one day.

4-hydroxybenzoic acid: 3-aminobenzamide (**23**): The two compounds were taken together in a mortar in 1:1 ratio and ground with a few drops of MeOH. The grinding process was repeated thrice. Solutions of resulting powder were kept for crystallizations. Diffraction quality crystals were obtained from MeOH after 3-4 days by solvent evaporation.

3-hydroxybenzoic acid: 3-aminobenzamide (**24**): The two compounds were taken together in a mortar in 1:1 ratio and ground with a few drops of MeOH. The grinding process was repeated thrice. Solutions of resulting powder were kept for crystallizations. Diffraction quality crystals were obtained from EtOH after 3-4 days by solvent evaporation.

3,5-dihydroxybenzoic acid: 4-aminobenzamide (**25**): The two compounds were taken together in a mortar in 1:1 ratio and ground with a few drops of MeOH. The grinding process was repeated

thrice. Solutions of resulting powder were kept for crystallizations. Diffraction quality crystals were obtained from EtOH after 3-4 days by solvent evaporation.

Table S1 Additional crystallographic details

Name	**11**	**12**	**13**	**14**	**15**
Molecular weight	325.00	325.00	359.44	359.44	358.44
ρ_{calc} (g/cm^3)	1.568	1.601	1.676	1.614	1.655
F(000)	328	656	720	1440	358
μ (MoK_α) (mm^{-1})	0.845	0.863	1.007	0.970	0.997
Temp. (K)	150	150	150	150	150
θ Range for data collection (°)	3.0, 27.5	3.1, 27.5	3.1, 27.5	3.0, 25.2	3.1, 27.5
R_1	0.0322	0.0330	0.0256	0.0925	0.0642
wR_2	0.0836	0.0846	0.0558	0.2015	0.1496
Goodness-of-fit	1.09	1.09	1.06	1.21	1.04
Reflns collected	7315	13875	15205	12389	7652
Unique reflns	3144	3066	6493	2662	3293
Observed reflns	2604	2703	6193	2516	2949

Name	**16**	**17**	**18**	**19**	**20**
Molecular weight	369.45	416.45	449.89	325.00	325.00
ρ_{calc} (g/cm^3)	1.753	1.909	1.991	1.626	1.624
F(000)	364	400	430	656	328
μ (MoK_α) (mm^{-1})	3.494	2.749	2.835	0.876	0.875
Temp. (K)	150	150	150	150	150
θ Range for data collection (°)	3.0, 27.5	3.2, 27.5	3.0, 27.5	2.2, 27.6	2.0, 25.2
R_1	0.0394	0.0276	0.0475	0.0416	0.0422
wR_2	0.0978	0.0697	0.1235	0.1436	0.1390
Goodness-of-fit	1.04	1.12	1.20	1.14	1.14
Reflns collected	7199	7553	7564	13867	5849
Unique reflns	3204	3313	3429	3068	2397
Observed reflns	2717	3105	3038	2843	2240

Name	**21**	**22**	**23**	**24**	**25**
Molecular weight	359.44	359.44	274.27	274.27	580.54
ρ_{calc} (g/cm^3)	1.624	1.673	1.418	1.430	1.458
F(000)	360	360	1152	576	304
μ (MoK_α) (mm^{-1})	0.975	1.005	0.106	0.106	0.112
Temp. (K)	150	150	150	150	150
θ Range for data collection (°)	3.0, 27.5	3.2, 27.5	3.3, 27.5	1.6, 27.5	3.3, 27.5
R_1	0.0371	0.0252	0.0385	0.0476	0.0402
wR_2	0.0992	0.0665	0.1085	0.1689	0.1083
Goodness-of-fit	1.09	1.07	1.10	1.14	1.03
Reflns collected	7780	7624	12832	12530	6973
Unique reflns	3366	3265	2944	2914	3013
Observed reflns	3035	3023	2635	2416	2472

S2. Details of database studies (CSD)

Table S2 Multi-component crystals: 29

**O–H···N**	**N–H···O**	**N–H···π**	**O–H···O**	**N–H···N**	**Synthon**	**Others**
CIQPAU	CIQPAU					
EXAMUM						
EXANAT						
EXAPID01			EXAPID01			
FIDLIO	FIDLIO				**II**	
FIDLOU	FIDLOU				**II**	
FIDLUA	FIDLUA				**I**	
FIDMAH	FIDMAH		FIDMAH	FIDMAH		
HEBHAK						
JAKPIV	JAKPIV				**III**	
JAKPOB	JAKPOB				**III**	
JAKPUH	JAKPUH				**III**	
KIBQOC			KIBQOC			N–H····F
KIBQOC01	KIBQOC01					O–H····F
OFEPUK	OFEPUK		OFEPUK			
OFEQIZ	OFEQIZ		OFEQIZ			OH: NH_2 (2:1)
PITYAS	PITYAS				**III**	
PITYEW	PITYEW				**III**	
PITYIA	PITYIA				**III**	
PITYOG	PITYOG				**III**	
PUVNID	PUVNID					Synthon is interrupted by H_2O
PUZZIT		PUZZIT				OH: NH_2 (2:1)

SARDAQ	SARDAQ				**III**	
SARDIY	SARDIY				**III**	
SARDOE	SARDOE				**III**	
SARLEC	SARLEC				**II**	NH_2: OH (2:1)
ZAQSOB	ZAQSOB					Interrupted
ZEHDUL	ZEHDUL	ZEHDUL	ZEHDUL			
ZEHFAT		ZEHFAT	ZEHFAT			

Table S3 Single-component crystals: 44

**O–H···N**	**N–H···O**	**N–H···π**	**O–H···O**	**N–H···N**	**Synthon**	**Others**
AMNPHA						Interrupted by NO_2
AMPHOL01	AMPHOL01				**III**	
AMPHOM02	AMPHOM02	AMPHOM02				
AMPHOM03	AMPHOM03	AMPHOM03				
EBIFOL	EBIFOL				**III**	
EBIFUR	EBIFUR				**III**	
ENALOU	ENALOU				**II**	
ENALUA	ENALUA				**III**	
ENAMAH	ENAMAH	ENAMAH				
ENAMEL	ENAMEL				**III**	
ENAMIP	ENAMIP	ENAMIP				
ENAMOV	ENAMOV				**II**	
ENAMUB	ENAMUB				**III**	
ENANAI	ENANAI				**III**	
ENANEM	ENANEM	ENANEM				
ENANIQ	ENANIQ	ENANIQ				

ENANOW	ENANOW				**II**	
ENANUL	ENANUL				**II**	
ENAPAK	ENAPAK	ENAPAK				
FUHTAE	FUHTAE				**I**	
FUHTAE01	FUHTAE01				**I**	
GEBVAK	GEBVAK	GEBVAK				
GEBVAK01	GEBVAK01	GEBVAK01				
GIVRIM		GIVRIM				
HIWMUW	HIWMUW	HIMUW				
HIWNAD	HIWNAD	HIWNAD				
HIWNEH						N–H⋯O=C
MAMPOL	MAMPOL	MAMPOL				
MAMPOL02	MAMPOL02	MAMPOL02				
MASZIQ	MASZIQ	MASZIQ				
MASZOW	MASZOW				**II**	
MASZUC	MASZUC	MASZUC				
MATBAL	MATBAL				**II**	
MATBEP	MATBEP	MATBEP				
MATBIT	MATBIT	MATBIT				
MATBOZ	MATBOZ	MATBOZ				
MATBUF	MATBUF				**II**	
MATCAM	MATCAM				**II**	
MATCEQ	MATCEQ				**II**	
NODTIJ	NODTIJ					Interrupted by NO_2
PEJCAJ	PEJCAJ					Not Infinite
PEJCAJ01	PEJCAJ01		PEJCAJ01	PEJCAJ01		

PITZAT	PITZAT				**III**	
QEPGAU	QEPGAU	QEPGAU				
SADJAK		SADJAK	SADJAK			
UHEVOT		UHEVOT				
WURNAZ		WURNAZ				
YEJPUA	YEJPUA				**I**	

Figure S1 Diagrams of multi-component crystals with ···O–H···N–H··· hydrogen bonding

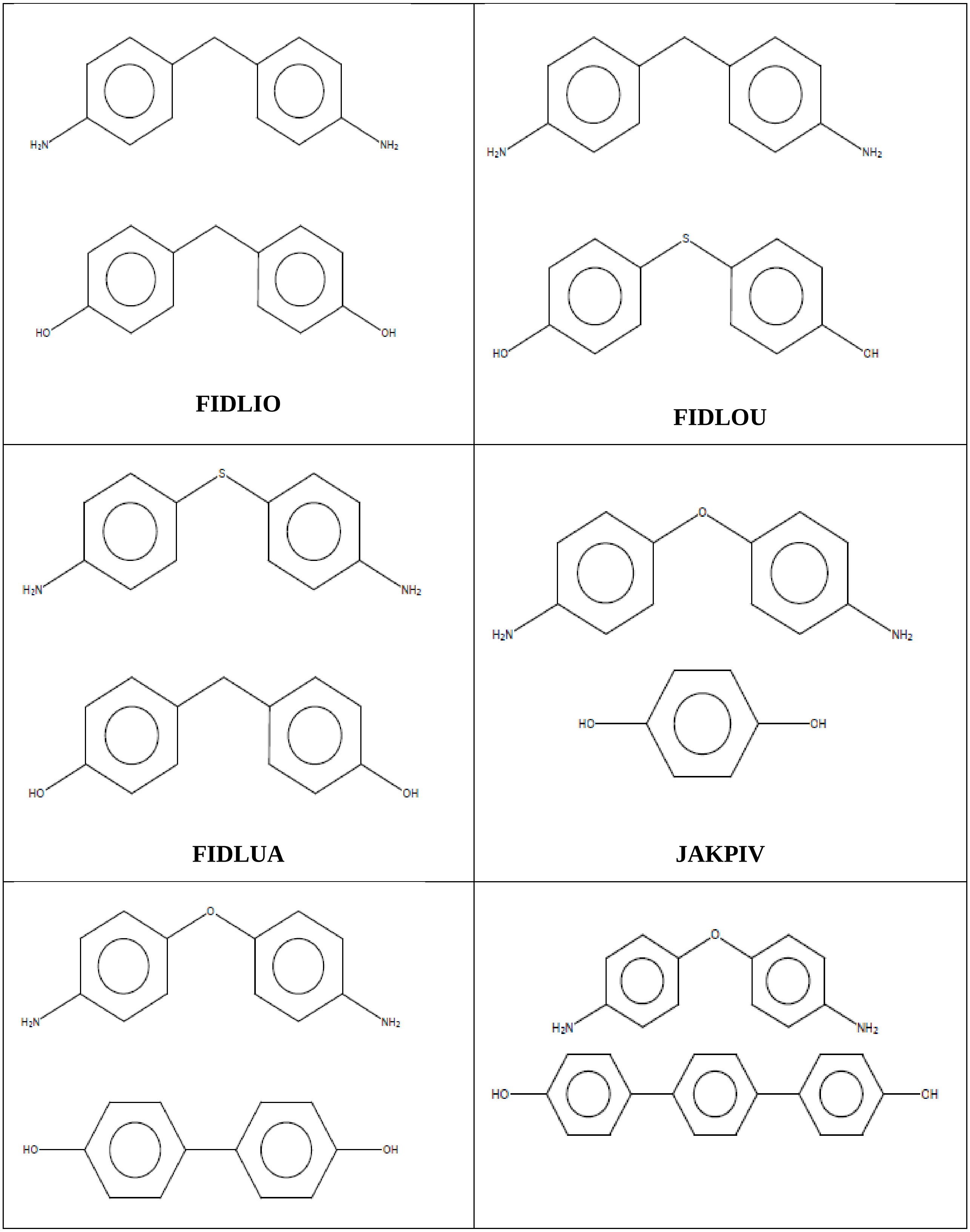

**JAKPOB**	**JAKPUH**
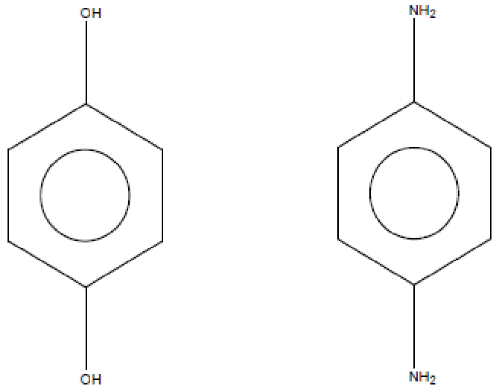<br>**PITWAS**	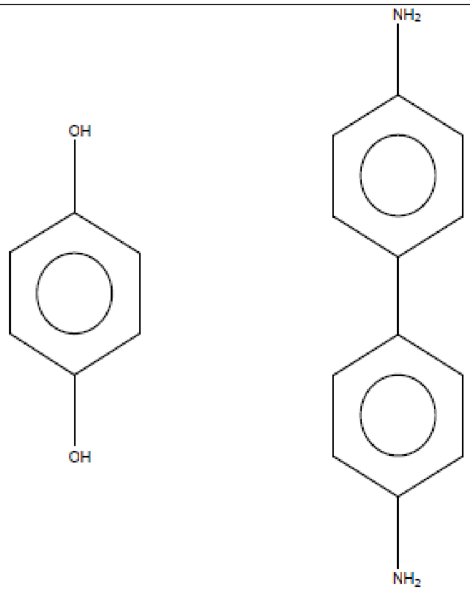<br>**PITYEW**
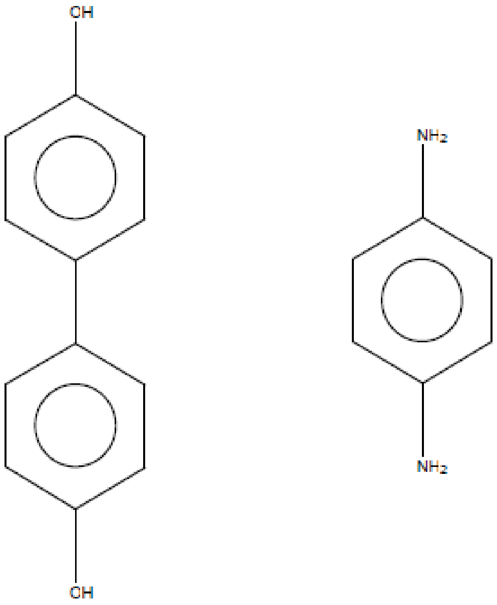<br>**PITYIA**	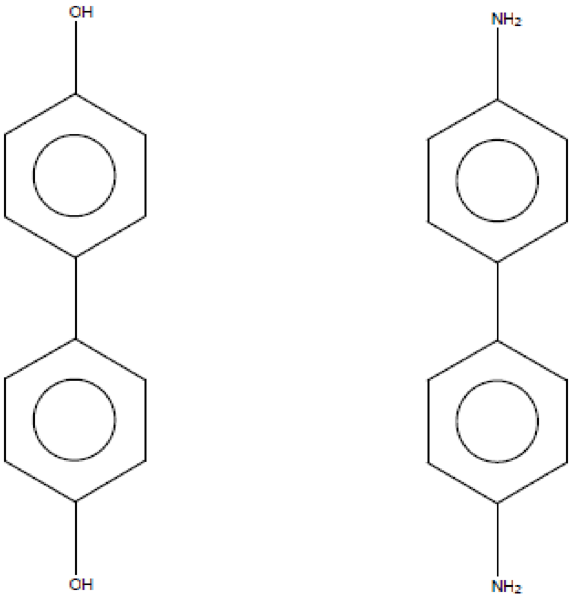<br>**PITYOG**

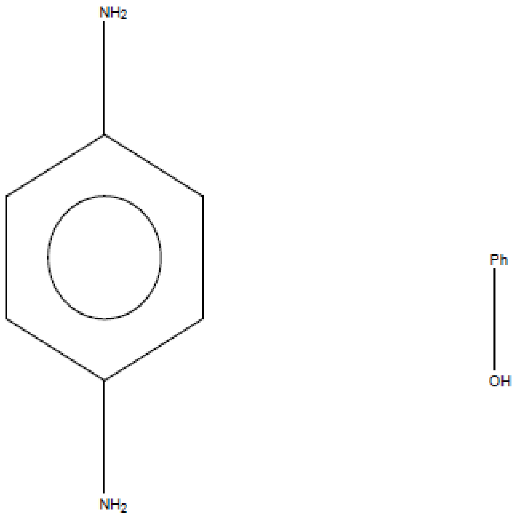

SARDAQ

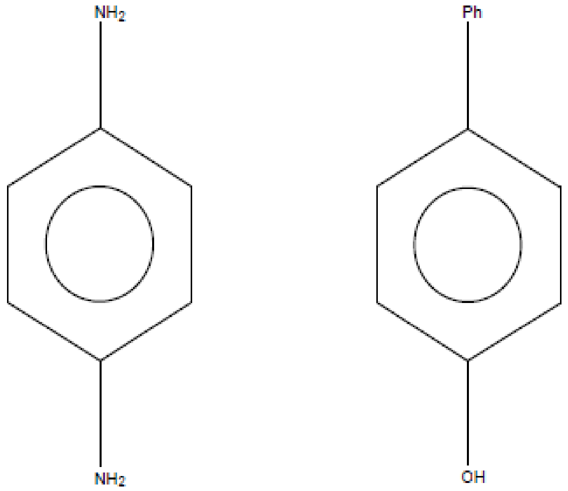

SARDIY

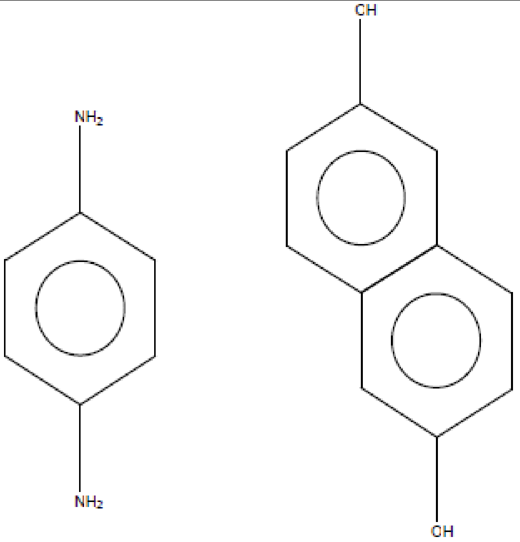

SARDOE

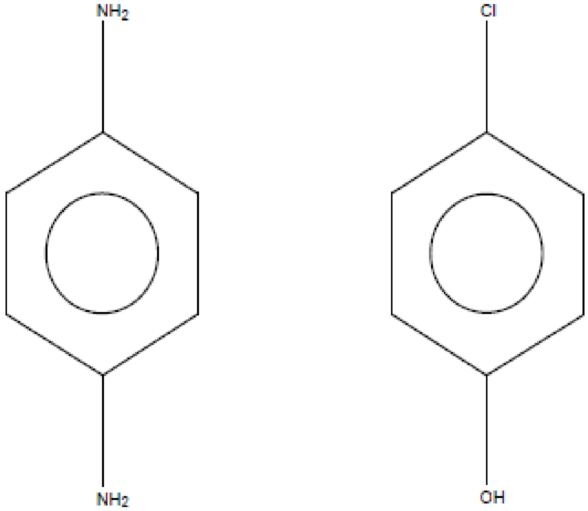

SARLEC

Table S4 Hydrogen-bonding parameters for cocrystals 11-25

	D–H···A	Symmetry code	D–H (Å)	H···A (Å)	D–H···A (∀)
**11**	N1–H1A···O1	-x,3-y,1-z	0.829(19)	2.36(2)	147.7(19)
	O1–H1O···N1		0.79(2)	1.98(2)	171(3)
**12**	N1–H1B···O1	-x,1-y,2-z	0.85(3)	2.24(2)	150(2)
	O1–H1O···N1	1+x,y,1+z	0.81(3)	1.96(3)	174(3)
**13**	N1–H1A···O1	1-x,1/2+y,-z	0.87(2)	2.17(2)	162(2)
	N1–H1B···Cl10		0.84(3)	2.61 (3)	109 (2)
	O1–H1O···N2	1+x,-1+y,z	0.73 (3)	2.12 (3)	163(3)
	N2–H2A···Cl7		0.81 (3)	2.62 (2)	110 (2)
	N2–H2B···O2	-x,1/2+y,-z	0.82 (3)	2.44 (3)	161 (3)
	O2–H2O ··N1		0.75 (3)	2.03 (3)	165 (3)
	C6–H6···Cl7	1+x,-1+y,z	0.93	2.79	160.00
	C8–H8···Cl9	1-x,-1/2+y,-z	0.93	2.79	157.00
**14**	N1–H1B···O1	x,1-y,1-z	0.89 (3)	2.18 (4)	145 (5)
	O1–H1O···N1	-x,y,1/2-z	0.85 (7)	1.96 (6)	169 (5)
**15**	N1–H1A···O1	1+x,-1+y,1+z	0.92 (7)	2.31 (7)	142 (6)
	O1–H7···N1	-x,2-y,3-z	0.81 (6)	1.97 (6)	171 (6)
**16**	O1–H1O···N1	-x,1-y,2-z	0.82 (4)	1.96 (4)	173 (4)
	N1–H2B···O1	1+x,-1+y,1+z	0.85 (5)	2.34 (4)	148 (4)
**17**	N1–H1A···O1	1-x,2-y,-z	0. 83 (4)	2.33 (4)	151 (4)
	O1–H1O···N1	x,1+y,z	0.8200	1.9600	169.00
**18**	N1–H1A···O1		0.93 (7)	2.27 (7)	148 (6)
	O1–H1O···N1	-x,2-y,-z	0.8200	1.9800	169.00
**19**	N1–H1A···O1		0.90 (7)	2.29 (7)	149 (6)]
	O1–H1O···N1	-x,2-y,-z	0.8200	1.9800	169.00
**20**	N1–H1A···O1	x,1+y,z	0.80 (3)	2.28 (3)	154 (3)
	O1–H1O···N1	-x,1-y,1-z	0.92 (3)	1.81 (3)	166 (3)
**21**	N1–H1B···O1	-x,-y,1-z	0.85 (3)	2.19 (3)	163 (3)
	O1–H1O···N1	1+x,y,-1+z	0.8200	1.9300	163.00
**22**	N1–H1A···O1	1+x,1+y,z	0.845(19)	2.242(19)	156.5(19)
	O1–H1O···N1	1-x,-y,1-z	0.83(2)	1.89(2)	171(2)
**23**	N1–H1B···O1	1-x,-1+y,1/2-z	0.92 (2)	2.16 (2)	156.17 (16)
	O1–H1O···N1	x,2-y,-1/2+z	0.92 (2)	1.88 (2)	166.7 (19)
	N2–H2A···O2	1/2-x,1/2+y,1/2-z	0.880 (18)	2.153 (18)	159.9 (15)
	N2–H2B···O2	x,1+y,z	0.92 (2)	2.091 (19)	166.8 (16)
	O3–H3O···O4	x,-1+y,z	0.91 (2)	1.69 (2)	170.7 (19)
	C6–H6···O4	1-x,y,1/2-z	0.9300	2.5200	152.00
	C10–H10···O2	1/2-x,1/2+y,1/2-z	0.9300	2.5700	159.00
	C14–H14···O4		0.9300	2.4200	100.00

**24**	N1–H1A···O4	x,-1+y,z	0.90 (3)	2.43 (3)	130 (2)
	N1–H1B···O3	-x,-1/2+y,1/2-z	0.84 (3)	2.07 (3)	169 (2)
	O1–H1O···N2		0.85 (3)	1.97 (3)	167 (2)
	N2–H2B···O1	1-x,-1-y,1-z	0.87 (3)	2.16 (3)	161 (2)
	O2–H2O···O4	-x,1/2+y,1/2-z	0.8200	1.8200	170.00
	C3–H3···O2	-x,1-y,1-z	0.9300	2.6000	137.00
**25**	N1–H1A···O1		0.90 (2)	2.40 (2)	142.0 (16)
	O1–H1O···N1	-1-x,-y,1-z	0.86 (2)	1.96 (2)	172 (2)
	N2–H2A···O4	-1-x,1-y,1-z	0.88 (2)	2.30 (2)	162.5 (18)
	N2–H2B···O5	-3-x,1-y,-z	0.898 (19)	2.002 (19)	176.4 (18)
	O2–H2O···O5	1+x,y,1+z	0.93 (2)	1.74 (2)	167 (2)
	O4–H4O···O3	1-x,1-y,1-z	1.27 (4)	1.35 (4)	175 (3)
	C3–H3···O3		0.9300	2.4100	100.00
	C10–H10···O5		0.9300	2.4700	100.00
	C13–H13···O3	-1+x,y,z	0.9300	2.5600	126.00

Figure S2 ORTEP diagrams for cocrystals 11-25

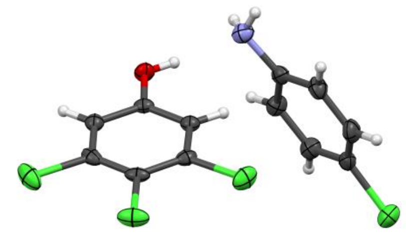	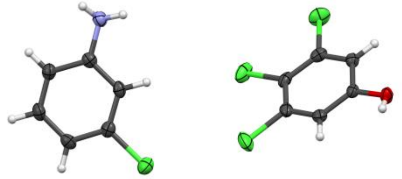	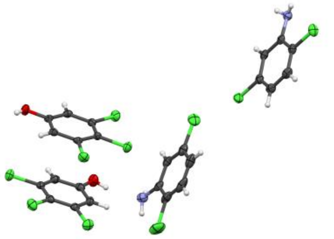
**11**	**12**	**13**
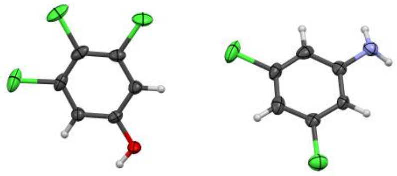	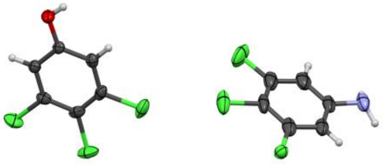	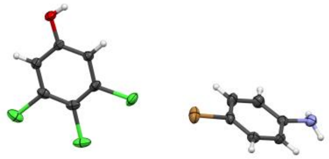
**14**	**15**	**16**
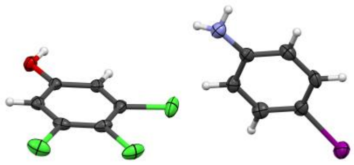	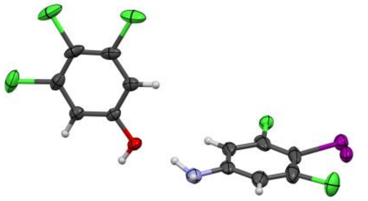	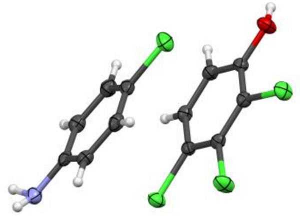
**17**	**18**	**19**

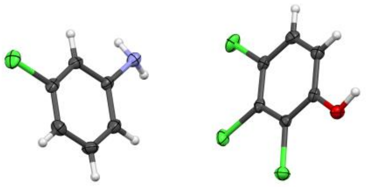	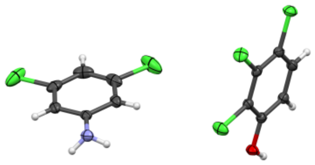	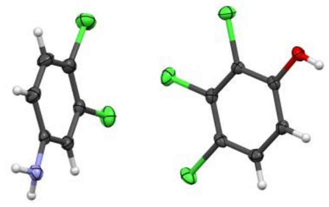
**20**	**21**	**22**
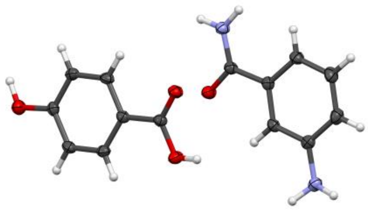	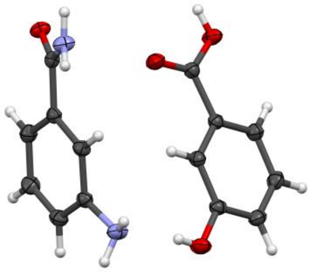	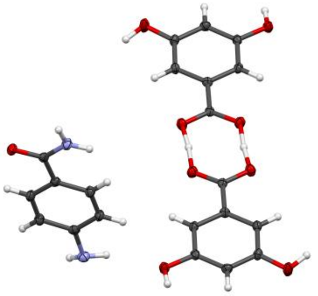
**23**	**24**	**25**

Figure S3 Packing diagram of 1,2,3-trichlorobenzene (TCBENZ)

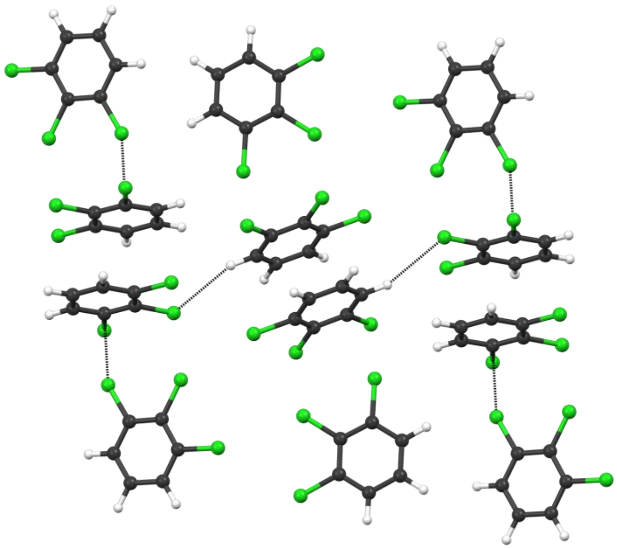

S3. NMR signal intensities as a function of gradient power

Figure S4 Representative spectra were obtained using the bipolar pulse pair gradient stimulated echo pulse program. Shown here are the spectra for sample **A** of **14**. See text for details.

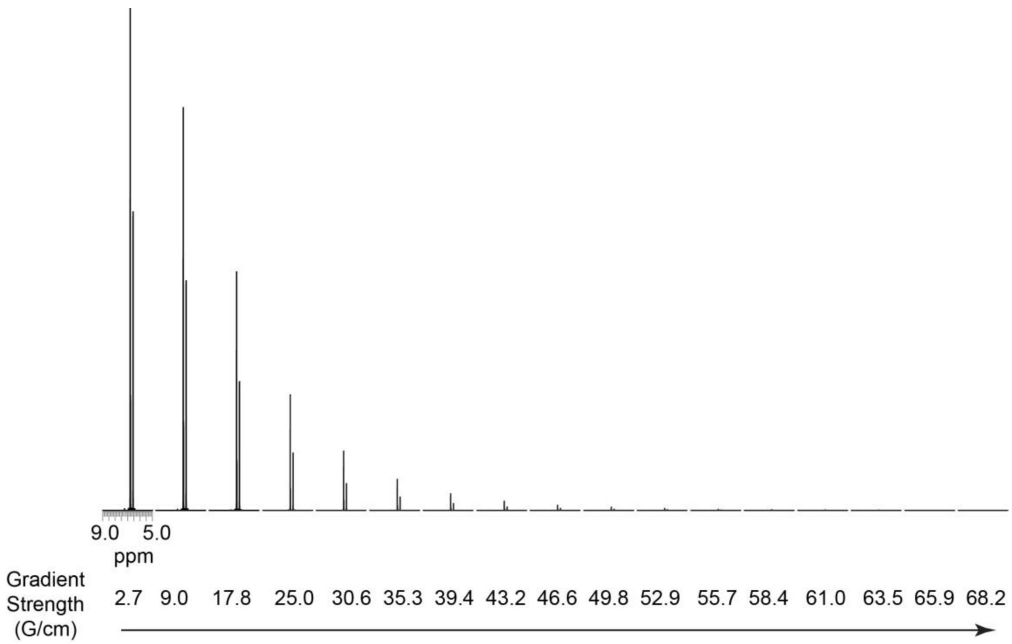

S4. 2-D DOSY plots

Figure S5 2-D DOSY plots of **A**, **E**, and **F** of **14** and **A_1**, **E_1** and **F_1** of **26**.

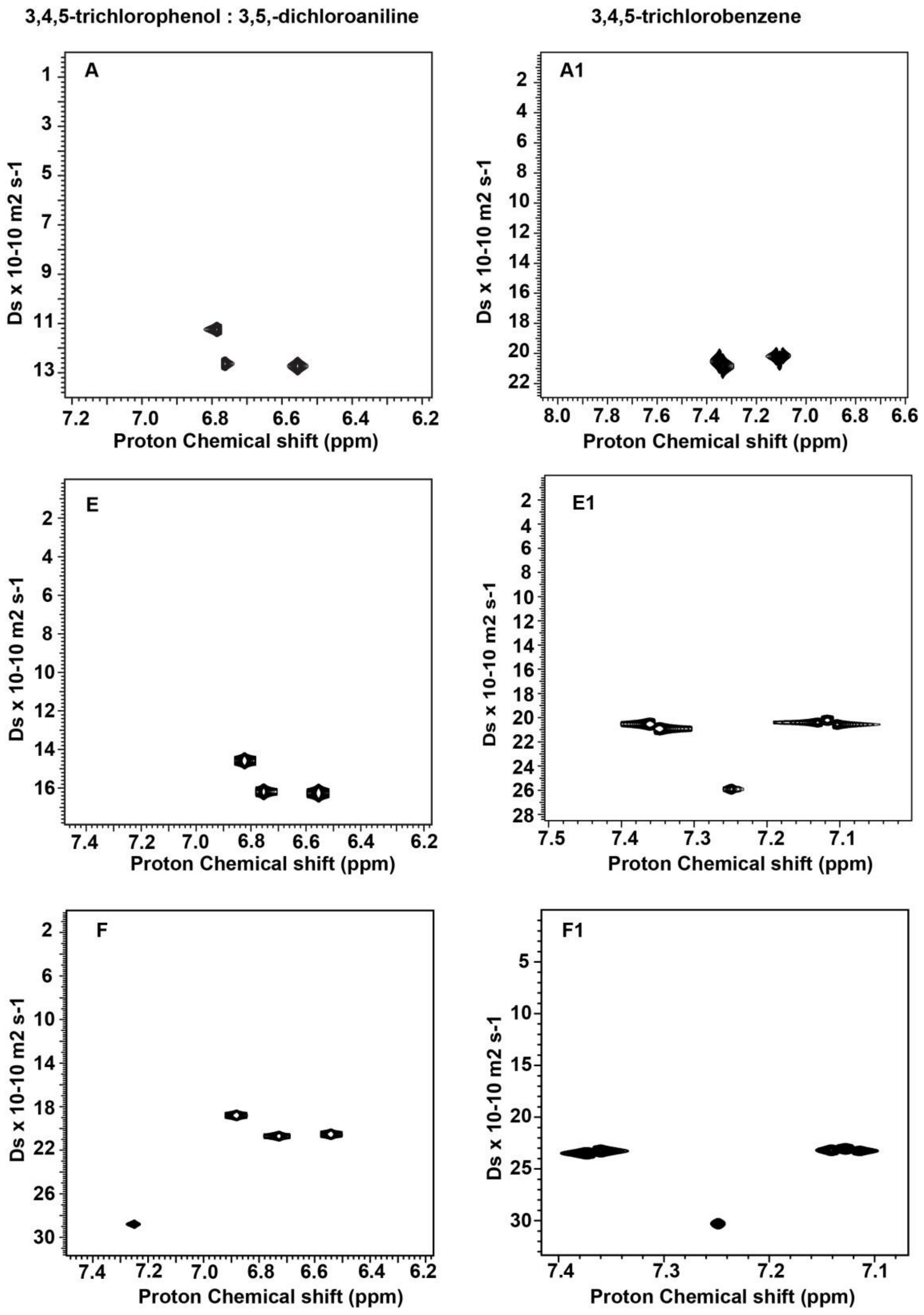

S5. ^{15}N NMR spectra for solutions A, E and F

Figure S6 One-dimensional ^{15}N spectra of samples **A** (black), **E** (green) and **F** (magenta). Also shown is the reference spectrum of ^{15}N enriched urea (red). The aniline N resonates at 58.7ppm. See text for details.

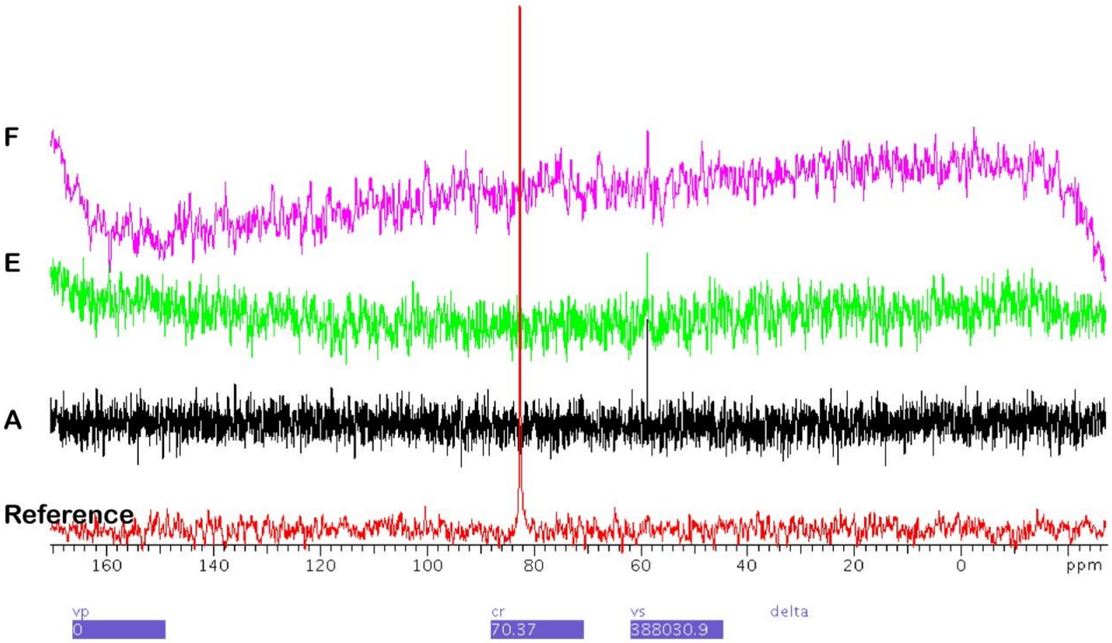

Supplement: Supplementary file 2 [file m-01-00228-sup2.pdf]
